# A 37 kb region upstream of brachyury comprising a notochord enhancer is essential for notochord and tail development

**DOI:** 10.1242/dev.200059

**Published:** 2021-12-15

**Authors:** Dennis Schifferl, Manuela Scholze-Wittler, Lars Wittler, Jesse V. Veenvliet, Frederic Koch, Bernhard G. Herrmann

**Affiliations:** 1 Max Planck Institute for Molecular Genetics, Department of Developmental Genetics, Ihnestr. 63-73, 14195 Berlin, Germany; 2 Institute of Biology, Department of Biology, Chemistry and Pharmacy, Freie Universität Berlin, Königin-Luise-Str. 1-3, 14195 Berlin, Germany

**Keywords:** Mouse, Embryo, Development, Notochord, Brachyury, Enhancer

## Abstract

The node-streak border region comprising notochord progenitor cells (NPCs) at the posterior node and neuro-mesodermal progenitor cells (NMPs) in the adjacent epiblast is the prime organizing center for axial elongation in mouse embryos. The T-box transcription factor brachyury (T) is essential for both formation of the notochord and maintenance of NMPs, and thus is a key regulator of trunk and tail development. The *T* promoter controlling *T* expression in NMPs and nascent mesoderm has been characterized in detail; however, control elements for *T* expression in the notochord have not been identified yet. We have generated a series of deletion alleles by CRISPR/Cas9 genome editing in mESCs, and analyzed their effects in mutant mouse embryos. We identified a 37 kb region upstream of *T* that is essential for notochord function and tailbud outgrowth. Within that region, we discovered a T-binding enhancer required for notochord cell specification and differentiation. Our data reveal a complex regulatory landscape controlling cell type-specific expression and function of *T* in NMP/nascent mesoderm and node/notochord, allowing proper trunk and tail development.

## Abstract (precis)

**Summary:** Genetic dissection of the mouse brachyury locus identifies a notochord enhancer and predicts additional control elements essential for trunk and tail development of the mouse embryo.

## INTRODUCTION

The mammalian embryo is generated in three consecutive phases, starting with head formation from the epiblast, continued by trunk development from the primitive streak acting as growth zone for posterior elongation, and finally tail development from the tailbud. The elongation process is driven by progenitor cells in the growth zone that continuously generate descendants added to the growing anterior-posterior axis (reviewed by Wilson et al., 2009; Wymeersch et al., 2021). Neuro-mesodermal progenitors (NMPs) located in the epiblast at the anterior end of the growth zone, termed node-streak border (NSB), give rise to neural and mesodermal tissues, and beneath, notochord progenitors provide descendants to the node and notochord. The node comprises the trunk organizer involved in medio-lateral patterning of nascent mesoderm (Beddington, 1994; Kinder et al., 2001; Wymeersch et al., 2016). The notochord acts as source of signals patterning the neighboring neural tube, paraxial mesoderm and gut (Stemple, 2005).

The T-box transcription factor brachyury (T) is a key regulator for multiple processes driving axis elongation in vertebrates (Gentsch et al., 2013; Herrmann et al., 1990; Martin and Kimelman, 2008; Stott et al., 1993). Homozygous *T^−/−^* mutant mouse embryos lack the node and trunk notochord, fail to form paraxial mesoderm in the trunk, and arrest axial elongation (Chesley, 1935; Gluecksohn-Schoenheimer, 1944). Heterozygous (*T^+/−^*) mouse mutants are viable, but develop short tails of variable length depending on the extent of the tail notochord (Dobrovolskaia-Zavadskaia, 1927; Gluecksohn-Schoenheimer, 1944).

T activity is essential for notochord formation, notochord differentiation and NMP maintenance (Cambray and Wilson, 2002, 2007; Henrique et al., 2015; Koch et al., 2017; Martin and Kimelman, 2010; Tsakiridis and Wilson, 2015; Tzouanacou et al., 2009). In NMPs, T and the signal molecule Wnt3a form a positive-feedback loop essential for axis elongation and mesodermal lineage choice, the latter in antagonism with the pro-neural activity of Sox2 (Garriock et al., 2015; Koch et al., 2017; Martin and Kimelman, 2008, 2012; Turner et al., 2014). As pan-mesodermal lineage control factor T plays an important role in remodeling the epigenome from the progenitor state to a mesodermal identity, and controls mesodermal transcription factors such as Tbx6 and Msgn1, which are essential for paraxial mesoderm differentiation (Chalamalasetty et al., 2014; Hofmann et al., 2004; Koch et al., 2017; Wittler et al., 2007). The formation and maintenance of the node and notochord require a high level of T expression (Pennimpede et al., 2012; Stemple, 2005; Zhu et al., 2016). The dual essential role of T in NMPs and node/notochord demonstrate that T is the central transcription factor coordinating the organization of the progenitors and their descendants shaping the trunk and tail.

The detailed analysis of *T* control in these processes promises deeper insight into the mechanisms controlling the NMP/NSB domain. T expression is maintained in NMPs and in the node and notochord, but is transient in nascent mesoderm due to repression by Tbx6 (Gouti et al., 2017; Koch et al., 2017). The *T*-streak promoter (from −500 bp to the TSS) is sufficient for *T* expression in nascent mesoderm, but not in the notochord (Clements et al., 1996; Perantoni et al., 2005), and responsive to Wnt signaling (Arnold et al., 2000; Yamaguchi et al., 1999). The control elements for node and notochord expression of *T* have not been identified yet.

Here, we have generated a series of *T* deletion alleles in mouse ESCs by CRISPR/Cas9 technology, and present a detailed analysis of mutant embryos. We show that a 37 kb region upstream of *T* is essential for notochord specification and tail bud outgrowth, and identify an enhancer that controls *T* expression in the notochord within that region.

## RESULTS AND DISCUSSION

**Fig. 1.**
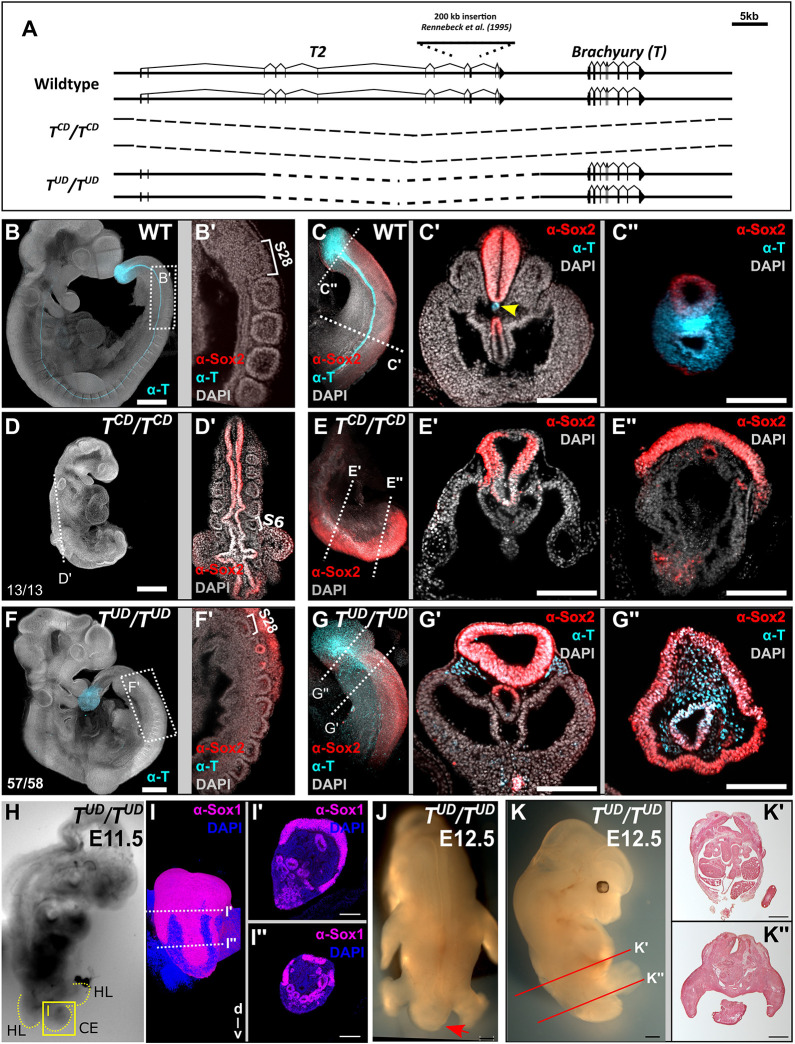
**A 37 kb upstream region contains regulatory elements essential for notochord formation and tailbud outgrowth.** (A) Schematic showing the murine *T2*-*T* locus. (B-G″) Immunostaining for T (cyan) and Sox2 (red) protein with nuclear DAPI (grey) staining in wild type (WT), *T* complete deletion (*T^CD^/T^CD^)* and *T* upstream deletion (*T^UD^/T^UD^*) E9-E9.75 embryos. (B,D,F) Maximum intensity projections of confocal stacks. Scale bars: 500 µm. (B′-C″,D′-E″,F′-G″,I′-I″) Optical sections with light sheet microscopy. Scale bars: 200 µm; number of somites formed is indicated (B′,D′,F′). The yellow arrowhead indicates the notochord (C′). (H) Bright-field image. CE, caudal end; HL, hindlimb. Yellow box marks the domain shown in I. (I-I″) Maximum intensity projection of stacks acquired by light sheet microscopy and single planes. (J) Dorsal view (red arrow indicates caudal end), (K) lateral view and (K′,K″) histological sections; axial levels are as indicated in K. Scale bars: 500 µm.

The activity of the *T* promoter in NMPs and nascent mesoderm, in combination with the failure of notochord formation in the *T^Bob^* mutant (Rennebeck et al., 1995) suggested that regulatory elements controlling *T* expression in the notochord are located far upstream of *T*. In search of such elements, we used CRISPR/Cas9 technology to generate deletion alleles in mESCs comprising the entire *T* region or a 37 kb upstream region (Fig. 1A), and analyzed their effects on embryonic development using the tetraploid complementation assay (Eakin and Hadjantonakis, 2006).

Immunofluorescent staining showed the expected expression of T in the notochord, NMPs and nascent mesoderm of wild-type embryos (Fig. 1B-C″, Wymeersch et al., 2016). A *T* deletion termed *T^CD^* spanning from −62 kb upstream to 10 kb downstream of *T*, including the *T* transcription unit, resulted in axial truncation and absence of the trunk notochord in homozygous embryos, as expected from the analysis of the original *T* mutant (Fig. 1D,D′, Chesley, 1935). The axial truncation phenotype is demonstrated by the depletion of NMPs and differentiation of their descendants into neural tissue at the expense of paraxial mesoderm (Koch et al., 2017), as visualized by an expansion of the neural plate identified by Sox2 protein, and lack of (pre-)somitic mesoderm posterior of the forelimb buds (Fig. 1E-E″).

Embryos carrying the 37 kb deletion, from −8.5 kb to −45.5 kb upstream of *T* comprising the *T^Bob^* integration site, (*T^UD^/T^UD^*) appeared almost normally developed at the early tailbud stage with fore- and hindlimb buds, somites and tailbud (Fig. 1F). Optical sectioning and antibody staining for T and Sox2, however, revealed major defects. Trunk somites appeared smaller than in wild-type embryos and malformed (Fig. 1F′). The notochord was missing in the mutants (Fig. 1F′-G′). In the caudal end and tailbud, paraxial mesoderm was strongly reduced, whereas neural tissue marked by Sox2 protein was largely expanded and surrounding the entire tailbud, which lacked notochordal cells (Fig. 1G′,G″). T expression was detected in cells of the tail gut and was stronger than in adjacent mesoderm. Sox1 staining of E11.5 *T^UD^/T^UD^* embryos showed massive expansion of neural tissue in the caudal end (Fig. 1H-I″, Fig. S1A-F), and histological sections from E12.5 embryos revealed major defects in neural tube and somite differentiation in the trunk (Fig. 1J-K″; Fig. S1G-L). The tail was not formed.

The data show that the 37 kb upstream region contains control elements essential for tailbud formation, tail outgrowth, and proper neural tube and somite differentiation, whereas T expression from the streak promoter is sufficient to support trunk formation from NMPs. The trunk phenotype can be attributed to the lack of signaling inputs from the missing notochord (Chiang et al., 1996). The failure of proper tailbud formation is explained by NMP differentiation towards neural tissue at the expense of mesoderm, accompanied by NMP loss preventing tail outgrowth.

**Fig. 2.**
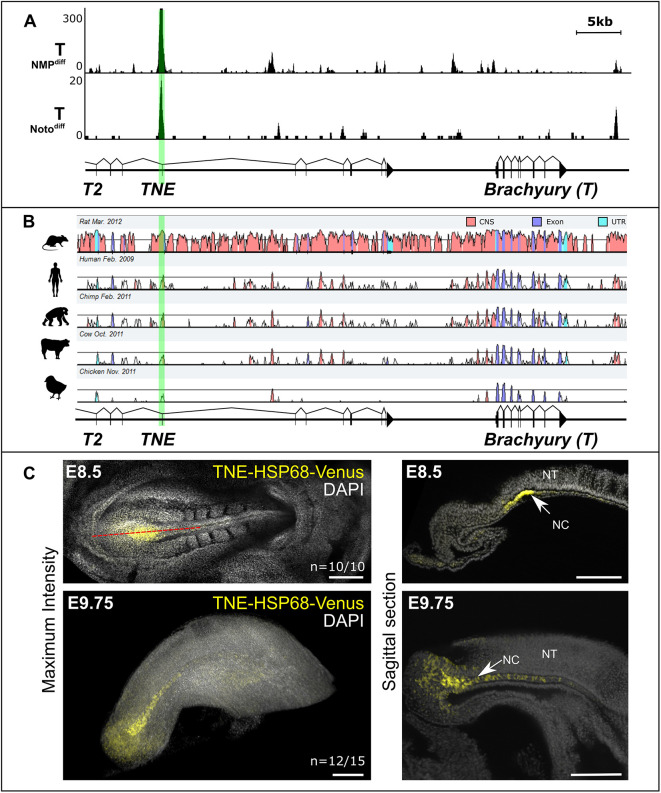
**A conserved T-bound genomic fragment shows enhancer activity in the notochord.** (A) ChIP-Seq tracks showing T peaks at the T locus in *in vitro*-derived NMPs (NMP^diff^; Koch et al., 2017) or notochord-like cells (Noto^diff^). The *TNE* region is highlighted in green. (B) Plot of corresponding genomic sequences in rat, human, chimp, cow and chicken against the mouse T locus (chr17:8,386,974-8,452,208; mm10). CNS, conserved non-coding sequence. (C) *TNE*-driven Venus reporter expression demonstrates enhancer activity in nascent axial mesoderm. Nuclei stained with DAPI (grey). Left: maximum intensity projections of confocal microscopy. Right: sagittal optical sections; light sheet acquisitions. NC, notochord; NT, neural tube. Scale bars: 200 µm.

Next, we searched for regulatory elements within the 37 kb region. As T expression in the notochord is maintained for several days, we suspected that T might be controlling its own expression in an autoregulatory manner via a notochord-specific enhancer. To search for T-binding sites in notochord precursors, we generated a *Noto::H2B-mCherry* (Noto^mC^) reporter construct by BAC recombineering, integrated the modified BAC into ES cells and validated proper reporter expression in transgenic embryos (Fig. S2). We then modified a protocol allowing differentiation of mESCs into Noto-positive cells *in vitro* (Winzi et al., 2011), generated Noto^mC^+ cells and performed ChIP-Seq using a T antibody (Fig. S3). The ChIP-Seq data identified a strong T peak about 38 kb upstream of the transcriptional start site of T (Fig. 2A). The peak position matches a strong T peak previously identified in NMPs (Koch et al., 2017) and coincides with exon 5 of *T2*. A genomic sequence comparison showed that the region of the peak is conserved in human, chimpanzee, cow and chick, suggesting that it may act as an enhancer (Fig. 2B).

We tested whether the T peak element is able to drive expression of a reporter in transgenic embryos. We cloned a 653 bp genomic fragment comprising the T peak region containing a palindromic T consensus binding site upstream of a HSP68 minimal promoter – Venus reporter (Fig. S4). The fragment also contains two consensus sites for Foxa2, another transcription factor essential for node and notochord formation (Ang and Rossant, 1994; Weinstein et al., 1994). We integrated the reporter construct into a *Rosa26*-close locus engineered for single copy integration using Cre recombinase in ES cells (Vidigal et al., 2010) and assayed mid-gestational embryos by fluorescence microscopy. The data show Venus expression primarily in the node and notochord at E8.5 and E9.75, confirming that the T peak element comprises a notochord enhancer bound by T (Fig. 2C). We therefore designated this element *TNE* (T-bound notochord enhancer).

**Fig. 3.**
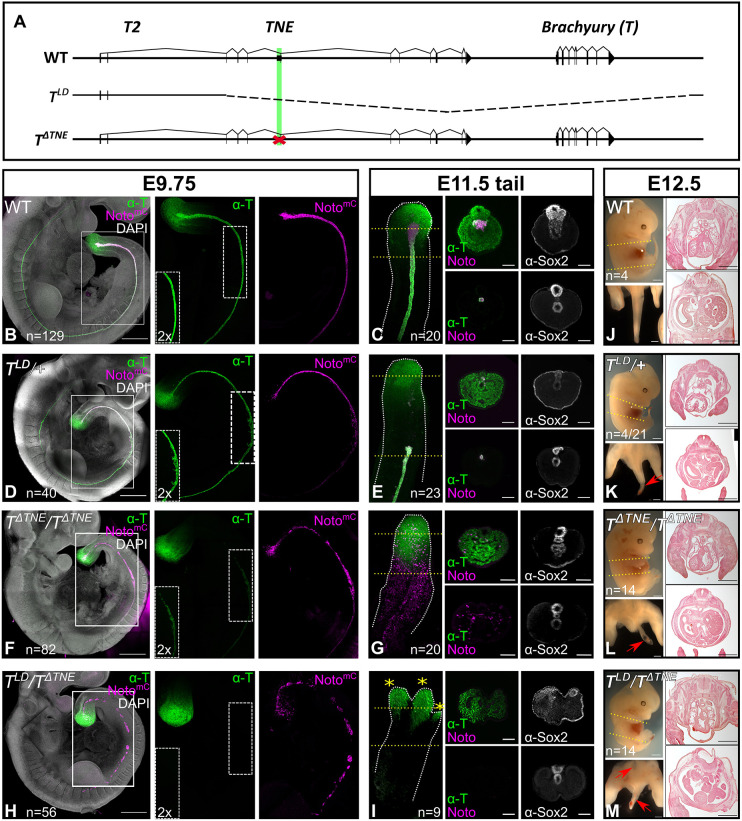
***TNE* is essential for brachyury expression in NPCs, and for notochord formation and differentiation.** (A) Schematic of deletion alleles. (B,D,F,H) Maximum intensity projections of E9.75 embryos with Noto^mC^ reporter signal, immunostaining for T (green) and DAPI nuclear staining (grey). Scale bars: 500 µm. The rectangle indicates the area magnified in single channels. 2× enhanced signal shown in the bottom left panel of the T channel. (C,E,G,I) Maximum intensity projections of E11.5 tails with immunostaining for T (green) and the Noto^mC^ reporter signal (magenta). Asterisks indicate bifurcations of the tailbud; yellow dashed lines indicate the position of optical sections: Scale bars: 100 µm. (J-M) Lateral and top views of E12.5 embryos, and histological transverse sections; section planes are indicated by yellow lines, defects in trunk (M) or tail (K,L,M) are indicated by red arrows. Scale bars: 500 µm. (D,E,K) *T^LD^/+* embryos showed a variable tail phenotype (11/23 had a truncated and 12/23 no tail notochord at E11.5; 17/21 were tailless and 4/21 were short tailed at E12.5); a short-tailed embryo is shown here.

Next, we genetically modified the T locus in the Noto^mC^ reporter ESC line using CRISPR/Cas9. We deleted *TNE* alone or in combination with a 63 kb genomic fragment spanning the *T* gene and upstream region (*T^LD^*) in ESCs (Fig. 3A; Fig. S5). We generated homozygous mutant embryos lacking *TNE* (*T^ΔTNE/ΔTNE^*), and analyzed the effect of *TNE* loss on notochord formation and embryogenesis in comparison with wild-type embryos, embryos heterozygous for *T^LD^* (*T^LD^*/+) or compound heterozygotes carrying *ΔTNE* opposite to *T^LD^* (*T^LD^/T^ΔTNE^*) (Fig. 3B-I; Fig. S5). At E9.75, wild-type embryos showed correct expression of the reporter in the node and notochord (Fig. 3B). At E11.5, mCherry expression in wild-type embryos was strongest in the caudal end of the tail notochord (Fig. 3C). E9.75 *T^LD^/+* embryos appeared normal, though malformed notochord was observed in the caudal trunk of some specimens (Fig. 3D). In contrast, in some E11.5 *T^LD^/+* embryos the tail notochord extended only half way into the tail, and the tailbud contained only very few Noto^mC^+ cells in the gut and ventral neural tube (Fig. 3E). These data show that notochord formation in *T^LD^/+* embryos was supported initially, but disrupted more posteriorly as notochord progenitors were incorrectly specified.

In E9.75 *T^ΔTNE^/T^ΔTNE^* embryos, T expression in the trunk notochord appeared weaker than in wild-type or *T^LD^/+* embryos, and an irregular trail of Noto^mC^+ cells lacking T protein extended into the tailbud (Fig. 3F). At E11.5, Noto^mC^+ cells of the tail region were widely dispersed, mostly in mesoderm, and not confined to the midline (Fig. 3G; Fig. S6). The tailbud contained few dispersed Noto^mC^+ cells, some in a tubular structure located between the neural tube and gut expressing Sox2 and Foxa2, but no T protein anterior to the tailbud (Fig. 3G; Fig. S6). The notochord was lacking, and tail outgrowth reached about half the normal length in these mutants.

The strongest phenotype was observed in *T^LD^/T^ΔTNE^* embryos (Fig. 3H,I). A notochord was not detectable in the trunk or in the tail, as demonstrated by T antibody staining. At E9.75, Noto^mC^+ cells devoid of T protein were detected in patches along the midline of the trunk and early tailbud (Fig. 3H). Noto^mC^+ cells were not detectable in the outgrowing tail at E11.5, which reached a length of about 10 somites (Fig. 3I). The tailbud was disorganized, splitting into two to three subdomains. A similar tail phenotype has been observed in *Gdf11*^−/−^ embryos showing almost complete absence of T in the tailbud notochord (Jurberg et al., 2013).

In all genotypes the tail outgrowths of E11.5 embryos showed T expression in the tailbud, and somite formation up to the point where the tailbud appeared malformed and further outgrowth started to fail. Differentiation was impaired in the entire region lacking a proper notochord, resulting in severe malformation of the posterior trunk (*T^LD^/T^ΔTNE^*) and/or degeneration of the tail (all mutants). Noto^mC^+ cells lacking T protein were not specified as notochord cells and instead contributed to neighboring tissues. *T^LD^/T^ΔTNE^* embryos generated only a few tail somites and the other mutants did not complete tail formation either. Both *T^LD^/T^ΔTNE^* and *T^ΔTNE^/T^ΔTNE^* mutants developed a tailless phenotype; the tail phenotype of *T^LD^/+* embryos varied from short tailed to tailless (Fig. 3J-M). Antibody staining for Casp3 revealed massive apoptosis (Fernandes-Alnemri et al., 1994) in the tailbud and tail somites of E11.5 *T^ΔTNE^/T^ΔTNE^* and *T^LD^/T^ΔTNE^* embryos lacking the notochord, thus providing a plausible explanation for the short-tailed or tailless phenotype (Fig. S7; Teillet et al., 1998).

To rule out the possibility that the *T^ΔTNE^/T^ΔTNE^* phenotype was due to the deletion of exon 5 of T2, we generated mutations in both alleles of exon 2, resulting in frame shifts of the predicted open reading frame ending in premature stop codons (Fig. S8). E12.5 embryos derived from mutant *T2^−/−^* ES cells showed no defect in tail or in trunk development, confirming that the *T^ΔTNE^/T^ΔTNE^* phenotype is not caused by the lack of T2, but due to the missing notochord enhancer.

**Fig. 4.**
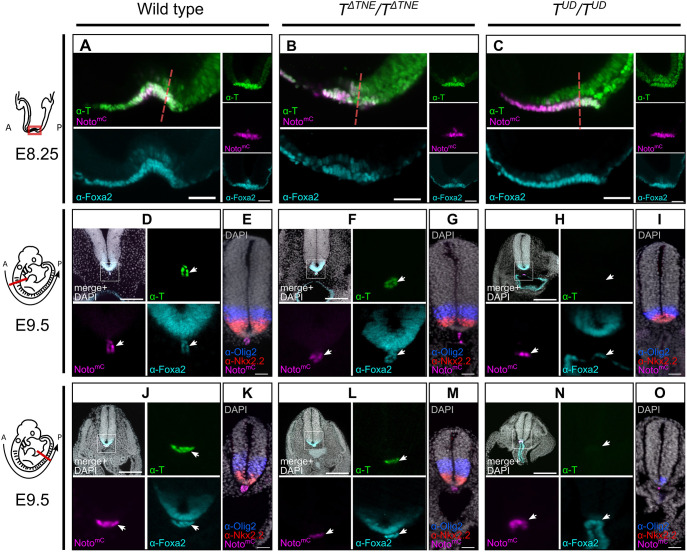
**Head process notochord is formed but not maintained in *T^UD^/T^UD^* mutants.** Light sheet micrographs of wild-type and mutant embryos with immunofluorescence for T (green) and Foxa2 (cyan) or Olig2 (blue) and Nkx2.2 (red). (A-C) Sagittal and transversal optical sections of the node at E8.25. Scale bars: 50 µm. (D-O) Transversal optical sections at different axial positions at E9.75, as indicated in the schematic on the left. (D-I) Cervical. (J-O) Lumbar. Scale bars: 200 µm in D,F,H,J,L,N; 50 µm in E,G,I,K,M,O. D,F,H,J,L,N and E,G,I,K,M,O show different embryos.

As formation of the head process notochord does not require T (Wilkinson et al., 1990; Yamanaka et al., 2007), we asked whether the *TNE* or *T^UD^* deletion affect notochord differentiation in the head and neck region. We found that the notochord expressing T, Foxa2 and Noto^mC^ had formed in both *T^ΔTNE^/T^ΔTNE^* and *T^UD^/T^UD^* mutant embryos at E8.25 (Fig. 4A-C). However, at E9.5 the notochord was missing from this region in *T^UD^/T^UD^* embryos, accompanied by integration of Noto^mC^+ cells into the foregut (Fig. 4D-I). Foxa2 protein was detected in the ventral neural tube, but the Olig2 and Nkx2.2 domains were ventrally shifted, suggesting impaired floor plate maintenance (Fig. 4H,I). Strong ventral neural tube patterning defects were detected in the trunk (Fig. 4J-O). *T^ΔTNE^/T^ΔTNE^* mutant embryos were not affected (Fig. 4F,G,L,M). The data suggest that *TNE* is not essential for head process notochord maintenance and differentiation, or for trunk notochord function.

The data show that *TNE* is a functional control element essential for T expression in the notochord, and for notochordal cell specification and differentiation. Reduced T expression in the trunk notochord of *T^ΔTNE^/T^ΔTNE^* mutants and absence of T expression and notochord in *T^LD^/T^ΔTNE^* and *T^UD^/T^UD^* embryos suggest the existence of another notochord enhancer (provisionally termed *NE2*) involved in notochord development, located in the region deleted in *T^UD^*. Loss of *TNE* can be compensated for in the head and neck region as well as in the trunk, but not in the tail, by *NE2*. A single copy of *NE2* in the absence of *TNE*, however, is not sufficient for notochord formation in the trunk or in the tail. Thus, the *T* gene dose and overall expression level in notochord cells are important for the phenotypic outcome. Strikingly, tail notochord formation and differentiation require two wild-type alleles of *T* in cis with *TNE* and possibly also *NE2*. The point where maximal *T* activity is required appears to coincide with the region where notochord progenitors need to expand in order to support proper tail notochord development and tail differentiation, i.e. in the posterior trunk (roughly at the lumbo-sacral transition; Yamanaka et al., 2007, Ukita et al., 2009). The variable tail length of *T^LD^/+* embryos suggests that the genetic background, and thus again the T expression level achieved by individual *T* alleles, can modulate the extension of the tail notochord.

The combined data suggest that the level of T expression in notochord progenitors in the posterior trunk might be related to the number of NPCs generated during expansion (Ukita et al., 2009; Wymeersch et al., 2019; Yamanaka et al., 2007), and thus the length of the tail, as tail differentiation is strongly dependent on notochord extension into the outgrowing tail. NMPs need much lower T expression levels than NPCs to function properly, and thus in *T* notochord enhancer mutants initial tail outgrowth can comprise multiple somite pairs. However, tailbud outgrowth beyond the notochord is limited, supporting the view that functional NPCs may form a niche for NMP maintenance (Edri et al., 2019; Wymeersch et al., 2019), although other explanations are conceivable.

## MATERIALS AND METHODS

### Generation of Noto reporter mESCs

A BAC containing ∼200 kb C57/BL6 genome surrounding the mouse *Noto* gene (RP23-289M19) was obtained from BACPAC resources. In order to engineer the Noto::H2B-mCherry reporter, a construct containing a H2B-fused fluorescent mCherry marker was inserted into the start codon of the gene via Red/ET recombineering (Muyrers et al., 1999). The reporter construct is followed by a *FRT*-site flanked selection cassette. The selection cassette consists of a *Pgk* promoter for expression in mESCs and an em7 promoter for selection in bacteria using the hygromycin resistance gene terminated by a *b-globin* polyadenylation signal.

In this study, male mESCs of the G4 hybrid line 129S6/SvEvTac×C57BL/6Ncr (George et al., 2007) served as the parental wild-type clone. All cell lines were regularly tested for possible mycoplasma contamination, using PCR Mycoplasma Test Kit II (Applichem A8994) according to the manufacturer's recommendations.

For random integration of the *Noto::H2B-mCherry* BAC, 5 µg of BAC DNA were linearized using PI-SceI (New England Biolabs R0696S) and electroporated into 3×10^6^ wild-type mESCs. Approximately 30 h after electroporation, selection was started applying 150 µg/ml hygromycin B (Merck 10843555001). Selection medium was refreshed daily until single colonies were clearly visible. Single clones were picked and genotyped by PCR. Oligonucleotides used in this study are listed in Table S1.

### Generation of deletion alleles

Vectors px335A_hCas9_D10A_G2P (a gift from Boris Greber, Max Planck Institute for Molecular Biomedicine, Münster, Germany) and px459-pSpCas9-2A-Puro (Addgene plasmid #48139) were used for the double nickase or conventional approach, respectively. Both vectors contain sequences encoding the *Streptococcus aureus* Cas9 enzyme controlled by a ubiquitous promoter, the guide RNA controlled by a human U6 promoter, a puromycin resistance gene for selection in ESCs and an ampicillin resistance for selection in bacteria. The CRISPR/Cas9 system was used for the introduction of targeted genomic deletions. Close to the desired break points, specific targeting sites 5′-*N_20_NGG*-3′ were identified and evaluated using the CRISPOR (http://crispor.tefor.net/crispor.py) tool. In cases where the first nucleotide of the *N_20_* targeting sequence was not a guanine, a guanine residue was added to the 5′end. For cloning, *BpiI* overhangs, 5′-CACC-3′ or 5′-AAAC-3′ were added to the target sequence or complementary strand sequence, respectively (Table S1).

Transfection of plasmids for CRISPR/Cas9 mediated deletions was performed using Lipofectamine 2000 reagent (Invitrogen 11668027). On the day before transfection, 3×10^5^ cells per well were seeded on gelatinized and wild-type feeder coated well of a six-well plate (Corning 3516). After overnight incubation, transfection mixes were prepared. For transfections in a six-well format, mixes of 125 µl Opti-MEM Reduced Serum Medium (Thermo Fisher 31985062) and 8 µg of each vector and 110 µl Opti-MEM and 25 µl Lipofectamine 2000 were prepared. 125 µl of each mix were combined, mixed well and incubated at room temperature for 15 min. Subsequently, 250 µl of transfection complex mix was diluted in 1.25 ml ES+LIF, added to the cells and incubated for 5 h. Finally, cells were trypsinized, split in 3:6, 2:6 and 1:6 ratios, and seeded on 6 cm dishes coated with puromycin resistant mEFs. 24 h post-transfection, transient selection was started applying 3 ml ES+LIF containing 2 µg/ml puromycin (Gibco 10130127) for 2 days and 3 ml ES+LIF containing 1 µg/ml puromycin (Gibco 10130127) for 1 day. After selection, ES+LIF medium was refreshed daily until colonies were clearly visible. Single clones were picked and screened by PCR, and verified by Sanger sequencing of purified PCR products extracted from agarose gels after electrophoresis. In the event that single PCR bands could not be separated by electrophoresis, fragments were cloned into pCR2.1 vectors using the reagents of a TA cloning kit (Invitrogen K202020) according to the manufacturer's procedure.

### Generation of enhancer reporter mESC lines

For recombinase-mediated cassette exchange, 3×10^5^ mESCs with a modified Rosa26 harboring locus (Vidigal et al., 2010) was co-transfected with 5 µg of linearized TNE-HSP68-Venus construct and 1 µg PGK-iCre vector using Lipofectamine 2000 as described above. For stable selection, cells were cultured in ES+LIF containing 350 µg/ml geneticin (Thermo Fisher 10131027).

### Generation of transgenic embryos

Transgenic mouse embryos were generated by diploid or tetraploid morula aggregation by the transgenic unit of the Max Planck Institute for Molecular Genetics in Berlin as described previously (Eakin and Hadjantonakis, 2006). All animal experiments were performed according to local animal welfare laws and approved by local authorities (covered by LaGeSo licenses G0243/18 and G0247/13).

### Embryo isolation

Timed pregnant foster mice were euthanized by carbon dioxide application and cervical dislocation. Embryos were isolated from uteri in 4°C pre-cooled PBS. After transfer to glass vials (Wheaton 224882), embryos were fixed in 4% paraformaldehyde (PFA)/PBS (Sigma Aldrich P6148). Fixation times were adapted to embryonic stage and subsequent procedures. For immunofluorescence, E6.5-E8.5 embryos were fixed for 40 min, E9.5-E10.5 for 1 h and E11.5 to E12.5 for 2 h. After fixation, embryos were washed three times with PBS and stored at 4°C until further processing.

### Whole-mount immunofluorescence and tissue clearing

If not specified otherwise, incubation in buffers was performed at room temperature on a roller. Embryos selected for immunofluorescence were collected in 4 ml glass vials (Wheaton 224882) and washed doe 3×10 min with PBS and for 3×10 min at room temperature with PBST (PBS containing 0.5% Triton X100, Merck 9002-93-1). For blocking, embryos were incubated in PBSTB (PBST containing 10% FBS) at 4°C for a minimum of 24 h. Primary antibody incubation was performed in PBSTB at 4°C for 48-96 h (antibodies are listed in Table S2). After incubation, remaining antibody solution was diluted by rinsing the samples three times with PBSTB followed by washing for 3×10 min with PBSTB and for 3×10 min in PBST. After washing, the specimens were incubated in PBSTB at 4°C overnight. Secondary antibody incubation was performed in PBSTB at 4°C for 24–48 h. Embryos were rinsed three times in PBSTB and washed for 2×20 min with PBSTB+0.02% DAPI (Roche Diagnostics 102362760019), for 3×20 min PBST+0.02% DAPI and transferred to eight-well glass bottom slides (Ibidi 80827). After additional washing steps in PBS for 3×10 min, embryos were either imaged or processed for tissue clearing.

For tissue clearing, stained embryos on eight-well glass slides were incubated in 0.02 M phosphate buffer (PB, 0.005 M NaH_2_PO_4_ and 0.015 M Na_2_HPO_4_, pH 7.4) at room temperature for 3×5 min. Before clearing, fresh refractive index matching solution (RIMS, 133% Histodenz, Sigma-Aldrich D2158) in 0.02 M PB was prepared and applied to the samples after careful removal of PB. Clearing was performed at 4°C on a shaking incubator for at least 24 h.

### Histology

PFA fixed E12.5 embryos were dehydrated through an ethanol series in 30%, 50% and 2×70% ethanol for 15 min each, processed in a MICROM STP 120 processor (Microm 813150) and embedded in paraffin wax (Leica 3801320) using an EC 350-1 embedding station (Microm). Sections of 10 µm thickness were prepared using a rotary microtome (Microm, HM355S), transferred onto adhesion microscope slides (Menzel K5800AMNZ72) and dried overnight at 37°C. Eosin (Merck 109844) counterstaining was performed according to standard procedures and specimens were mounted in Enthellan (Sigma-Aldrich 107960). Sections were imaged using an AxioZoom V16 stereomicroscope (Zeiss).

### Microscopy

Embryos were imaged using a Zeiss LSM880 laser scanning microscope with Airyscan detector or Zeiss Light sheet LS Z1 with appropriate filters for mCherry, Venus, DAPI, Alexa488 and Alexa647. For light-sheet microscopy, specimens were cleared and embedded in 1.5% low melting agarose (Sigma-Aldrich A9414)/PBS. Agarose columns containing the samples were inserted into the RIMS filled acquisition chamber and cleared for an additional 5 h to overnight depending on tissue volume. Post-acquisition processing was performed using ZEN Blue/Black (Zeiss) software or Arivis Vision 4D (Arivis).

### *In vitro* differentiation of Noto^mC^ cells

*In vitro* generation of notochord cells was performed using a modification of a previously published protocol (Winzi et al., 2011). Embryonic stem cells were seeded on 6 cm plates and passaged two times until about 70% confluence. Cells were trypsinized and resuspended in 2 ml ES+LIF. Feeder cells were depleted from single cell suspensions by sequential plating on 0.1% gelatin (Sigma G1393)-coated six-well plates (Corning 3335) in 25 min, 20 min and 15 min intervals. After feeder freeing, cells were resuspended in 1 ml NotoDiff medium (Knock Out Knockout Dulbecco's Modified Eagle's Medium with 100 µM sodium pyruvate (Gibco 10829-018), 1×N-2 Supplement (Gibco 17502-048), 1×B-27 Supplement w/o Vitamin A (Gibco 12587-010), 1×MEM non-essential amino acids (Gibco 1140-35), 5 µg/ml penicillin/streptomycin (Lonza DE17-603E), 100 µM β-mercaptothanol (Gibco 21985-023), 200 µM Glutamine (Lonza BE17-605E), counted and seeded on 0.1% gelatin (Sigma Aldrich G1393)-coated Nunclon Delta Surface 12-well plates (Thermo Scientific 150628) at a density of 5000 cells per well and per ml medium. During the 7 day differentiation protocol, medium was refreshed every 24 h. Cells were cultured in NotoDiff containing 1 ng/ml activin A (R&D Systems 338-AC) for 72 h. Subsequently, NotoDiff medium containing 1 ng/ml activin A (R&D Systems 338-AC), 100 ng/ml FGF2 (Peprotech 100-18B), 50 ng/ml Noggin (Peprotech 250-38), 1 µM AGN (Santa Cruz 193109) and 0.5 µM Smoothened agonist (Merck 364590-63-6) was applied for another 96 h.

### ChIP-Seq

For the identification of putative notochord enhancers, chromatin immunoprecipitation (ChIP) for T was performed using Noto-differentiated cells at D7, following a previously published protocol (Koch et al., 2011). ChIP-Seq sequencing libraries were generated using the TrueSeq ChIP-Seq kit (Ilumina) following the manufacturer's instructions with minor modifications. After adapter ligation, 0.95× of AMPure XP beads (Beckman Coulter A63880) were used for a single purification and the DNA was eluted using 15 µl of resuspension buffer (RSB, Illumina). After the addition of 1 µl primer mix (25 mM each: Primer 1, 5′-AATGATACGGCGACCACCGA*G-3′; and Primer 2, 5′-CAAGCAGAAGACGGCATACGA*G-3′) and 15 µl 2× Kapa HiFi HotStart Ready Mix (Kapa Biosystems), amplification was performed for 45 s at 98°C, five cycles of [15 s at 98°C, 30 s at 63°C and 30 s at 72°C] and a final 1 min incubation at 72°C. The PCR products were purified using 0.95× of beads and eluted using 21 µl of RSB. Libraries were directly amplified for additional 13 cycles and purified using AMPure XP beads. The libraries were quantified using the Qubit DNA HS assay and the library size was validated using DNA HS bioanalyzer chips (Agilent 5067-4626).

Reads were mapped to chromosomes 1-19, X, Y and M of the mouse mm10 genome using bowtie version 1.1.2 (Langmead et al., 2009), providing the options ‘-y -m 1 -S -I 100 -X 500’. The mapping information of the paired-end reads was used to elongate each fragment to its original size using a custom pearl script, with the result stored as a BED file. Reads were then sorted and deduplicated such that only one fragment with the same starting and end position was retained. For visualization, wiggle files were generated with BEDTools version 2.23.0 (Quinlan and Hall, 2010), converted to bigwig format and analyzed in the Integrated Genome Browser (Freese et al., 2016).

## Supplementary Material

Supplementary information

Reviewer comments
