# Supplementary material for: A 37 kb region upstream of brachyury comprising a notochord enhancer is essential for notochord and tail development
Source: Development. 2021 Dec 15;148(23):dev200059. doi: 10.1242/dev.200059 (PMC8722351; doi:10.1242/dev.200059)
Supplement: Supplementary information [file develop-148-200059-s1.pdf]

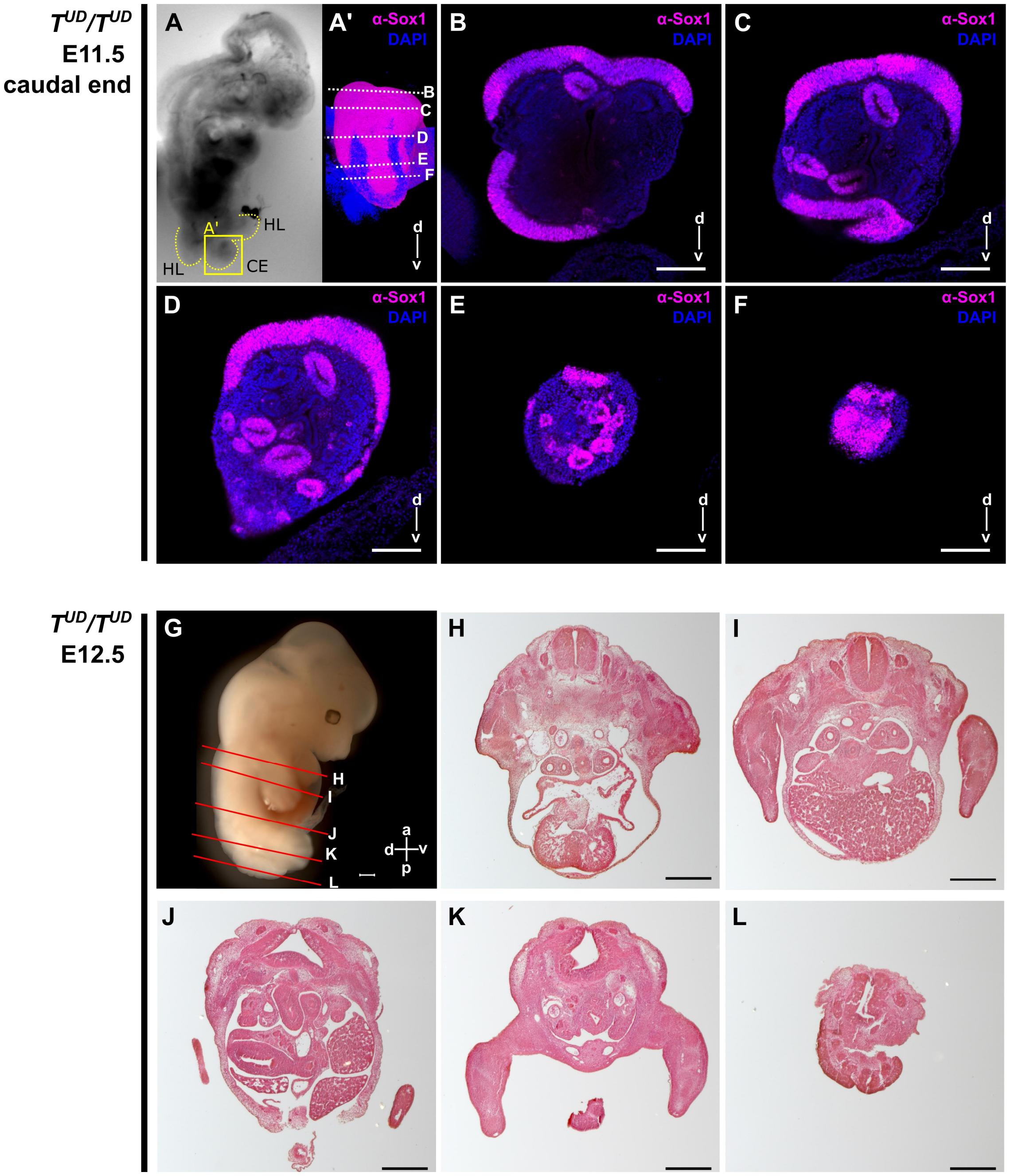

Fig. S1. Excess neural tissue is formed in the caudal end of T^{UD}/ T^{UD} mutants

(A) Bright field image of a cleared T^{UD}/ T^{UD} mutant embryo. HL = Hind limb. CE = Caudal End. **(A')** Maximum intensity projection of Light sheet micrographs from the caudal end region indicated by the yellow box in (A). White lines indicate the positions of optical transversal sections shown in (B-F). **(B-F)** Caudal tissue with immunofluorescence for Sox1 marking neural tissue. Transversal optical sections acquired by Light Sheet microscopy. Scale bar = 200 µm **(G)**: Lateral view of a E12.5 T^{UD}/ T^{UD} mutant embryo. Red lines indicate the section planes. **(H-L)**: Histological transversal sections demonstrating massive morphological disorganization, in particular of the spinal cord (J-L). Scale bar = 500 µm. a = anterior. p = posterior. d = dorsal. v = ventral.

Development • Supplementary information

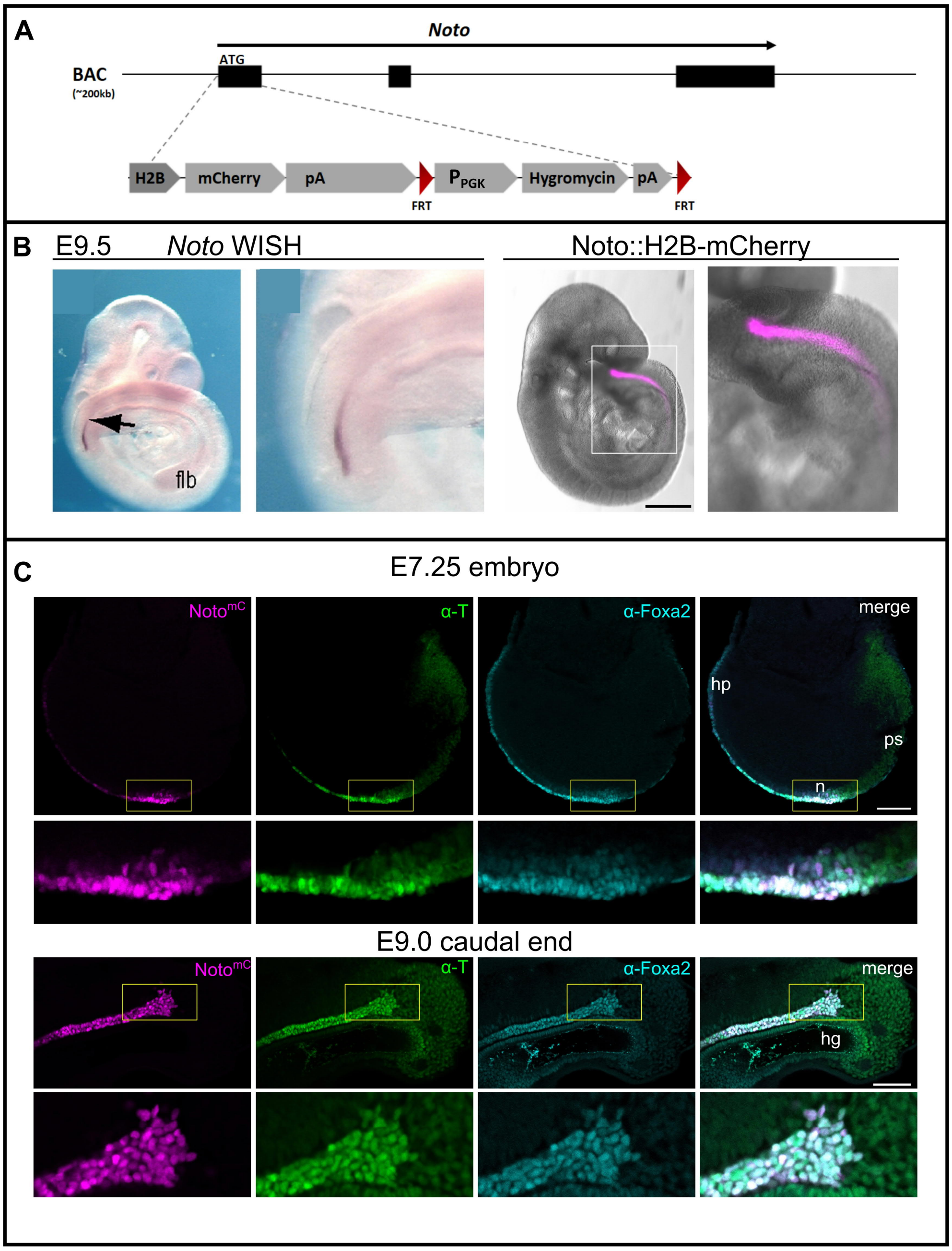

Fig. S2. Establishment of a Noto reporter mESC line

(A) Schematic representation of the *Noto::H2B-mCherry* reporter BAC. **(B)** Left: *Noto* whole mount *in situ* hybridization adapted from Pennimpede et al, 2012. flb = forelimb bud. Right: Noto^{mC} reporter fluorescence in a E9.5 embryo acquired by stereomicroscopy. Scale bar = 500 µm. **(C)** Confocal fluorescence microscopy of a E7.25 early headfold stage embryo and a E9.0 caudal end with Noto^{mC} reporter expression (magenta) and immunofluorescence for T (green) and Foxa2 (cyan). At E7.25, Noto^{mC} can be detected in the ventral node, head process notochord, crown cells and cells in the midline of the epiblast. At E9.0. Noto^{mC} marks the posterior end of the notochord. Scale bar = 100 µm. hp = head process. n = node. ps = primitive streak. hg = hindgut.

Development • Supplementary information

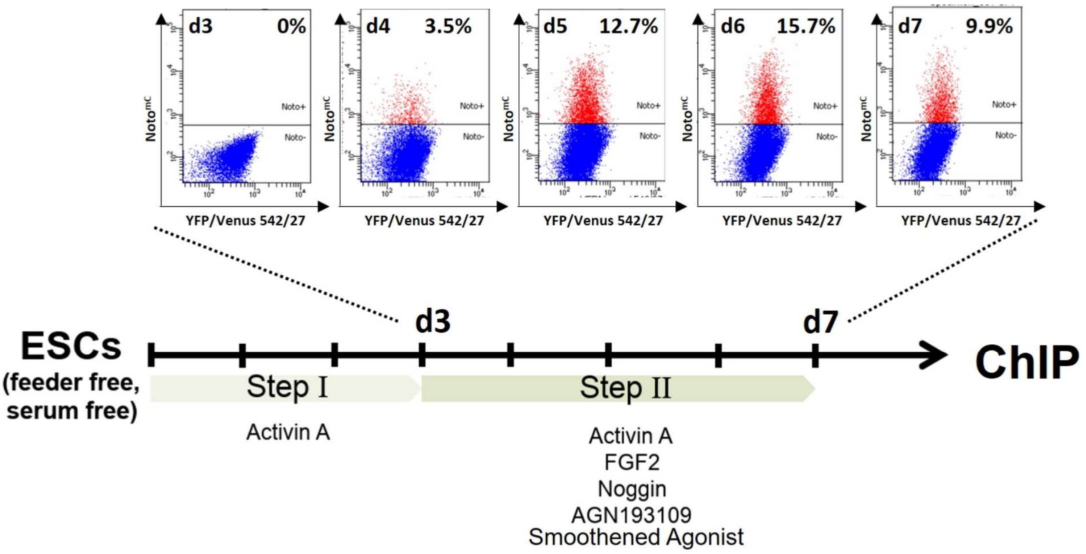

Fig. S3. Schematic representation of the notochord differentiation procedure

FACS plots show the proportion of Noto^{mC} expressing cells after application of step II medium at d3 in an exemplary experiment. For transcription factor ChIP-Seq, bulk cells were harvested between d6 and d7 of the procedure.

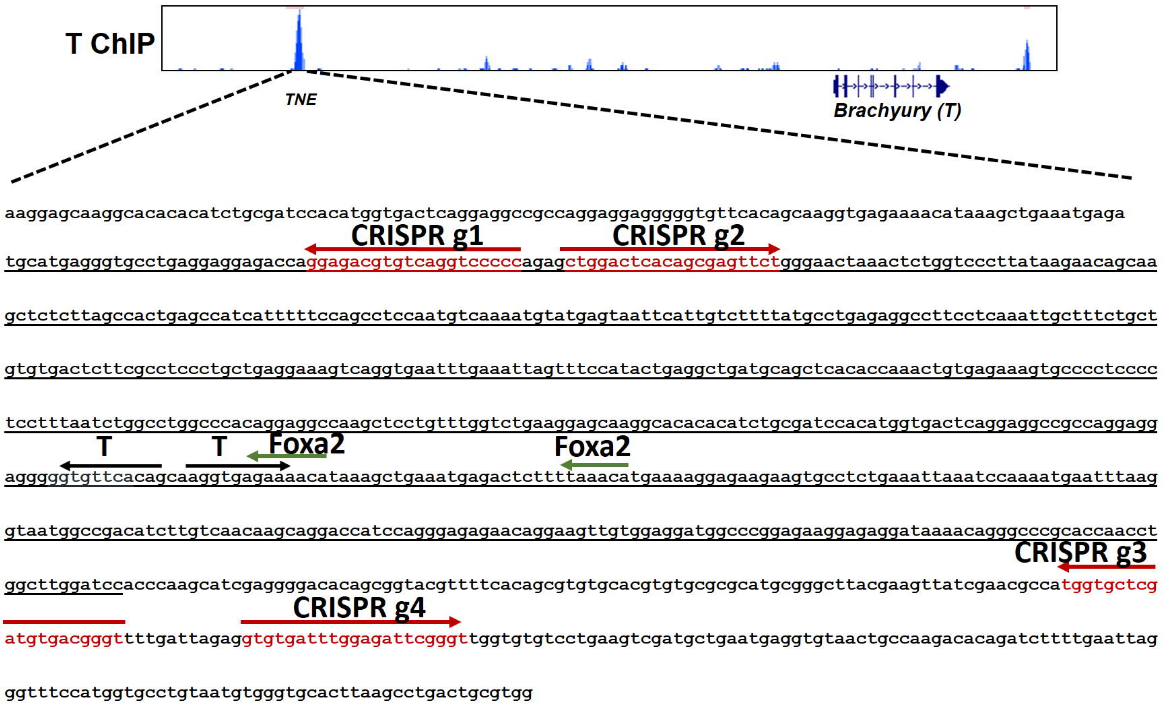

Fig. S4. *TNE* sequence

The sequence of the fragment tested in enhancer activity assays is underlined. Red letters and arrows indicate the guide RNA sequences used for CRISPR/Cas9 mediated deletions. Core binding motifs for T (black, palindromic consensus binding site) and Foxa2 (green) are highlighted. The ChIP-Seq track shows T binding sites in Noto^{diff} cells (this study).

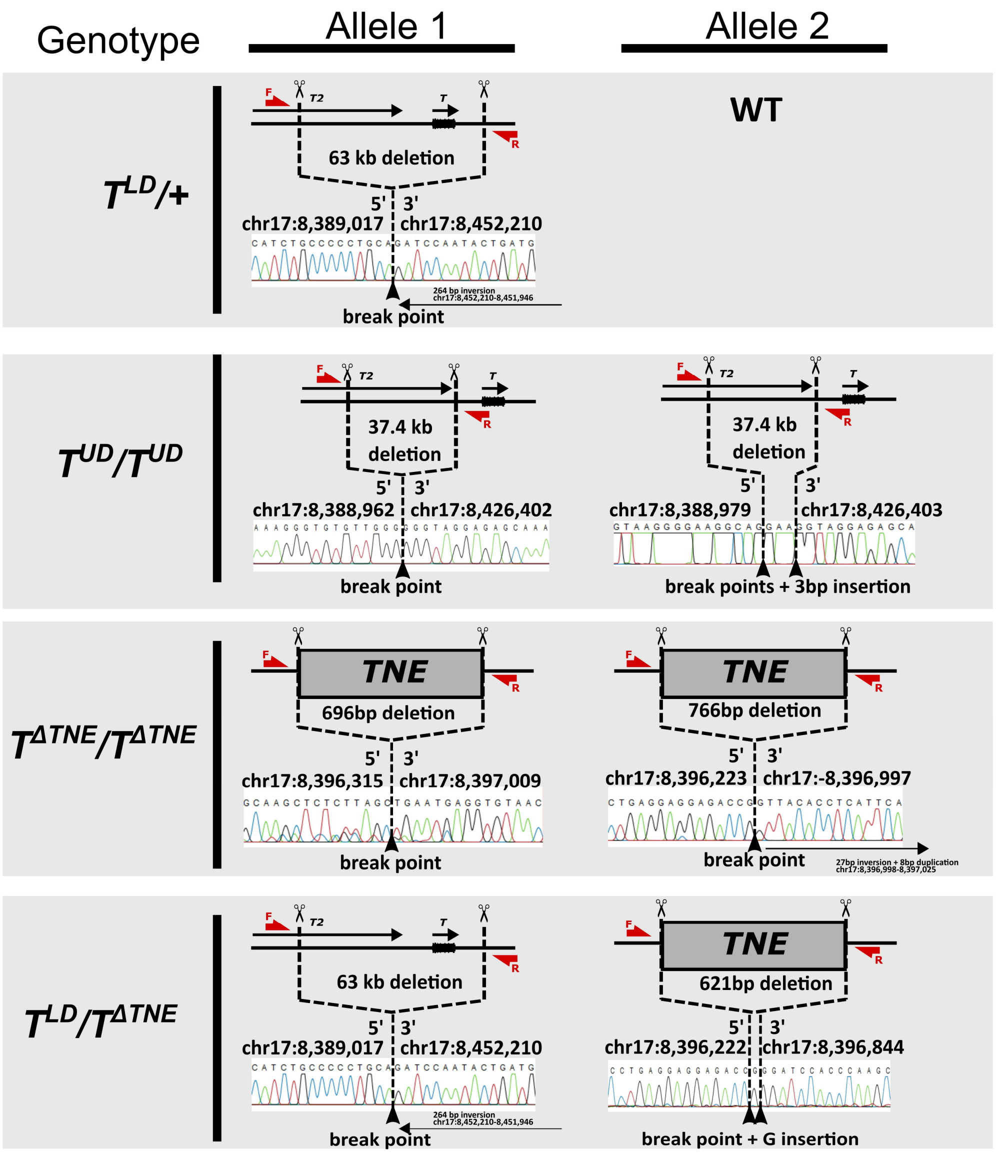

Fig. S5. Genotyping of Deletions

Red arrows indicate the position of the forward (F) and reverse (R) primers used for genotyping by PCR, not to scale. Scissors icons indicate the approximate positions of gRNA target sites. The dotted lines converging at the break point(s) mark the CRISPR/Cas9 mediated deletions. The coordinates (mm10) of the bordering 5' and 3' edges of the respective deletions are specified next to the break point(s). Sanger sequencing tracks of enhancer mutant PCR fragment or subcloned PCR fragments in case of double deletions show the region flanking the deletions. For each genotype at least two clones were generated and checked for phenotypic identity.

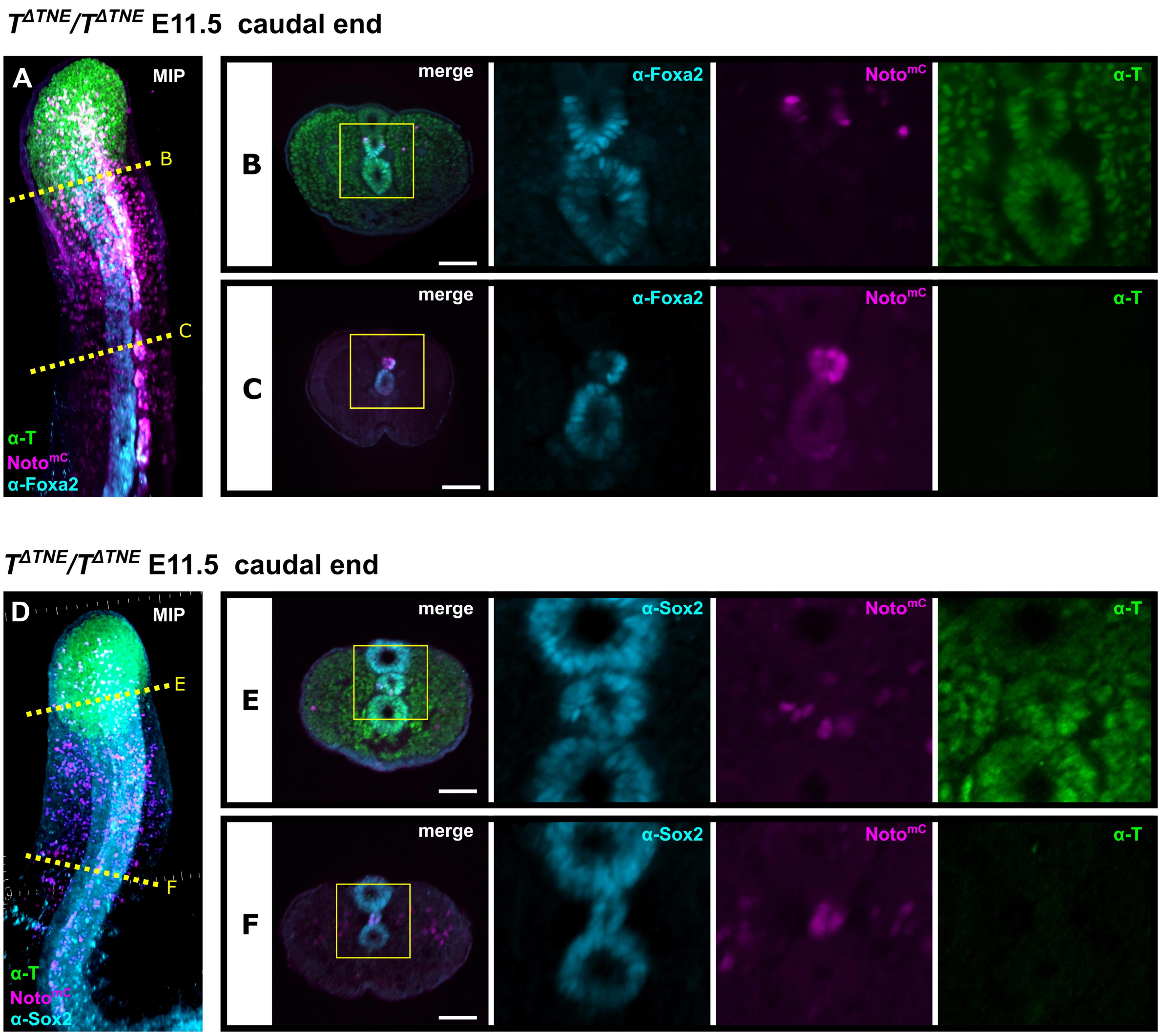

Fig. S6. Notochord progenitor cells in $T^{\Delta TNE}/T^{\Delta TNE}$ tailbuds are mal-specified and contribute to paraxial mesoderm and ectopic endodermal or ventral neural tube-like structures

(A) Maximum intensity projection of Light sheet micrographs of E11.5 tailbud with immunofluorescence for Foxa2 (cyan) and T (green). Yellow lines indicate the positions of optical sections shown in (B,C). **(B-C)** Transversal optical sections acquired by Light Sheet microscopy. Scale bar = 100 µm **(D)** Maximum intensity projection of Light Sheet micrographs of E11.5 tailbud with immunofluorescence for Sox2 (cyan) and T (green). Yellow lines indicate the positions of optical sections shown in (B,C). **(E-F)** Transversal optical sections acquired by Light Sheet microscopy. Scale bar = 100 µm. Noto^{mC} cells mostly contribute to paraxial mesoderm. An ectopic tubular midline structure formed between the neural tube and the gut expresses T, Sox2 and Foxa2 in the tailbud, but only Sox2 and Foxa2 anterior to the tailbud, indicating that it does not have notochordal identity. The presence of Noto^{mC} cells, however, points to its (at least partial) derivation from mal-specified notochord progenitor cells. The expression of Sox2 and Foxa2 suggests that it has acquired either endodermal or ventral neural tube-like identity. Ectopic neural tube-like structures have previously been described in the posterior trunk of Shh-Cre;T-shRNA T-knockdown embryos (Zhu et al. 2016).

Development • Supplementary information

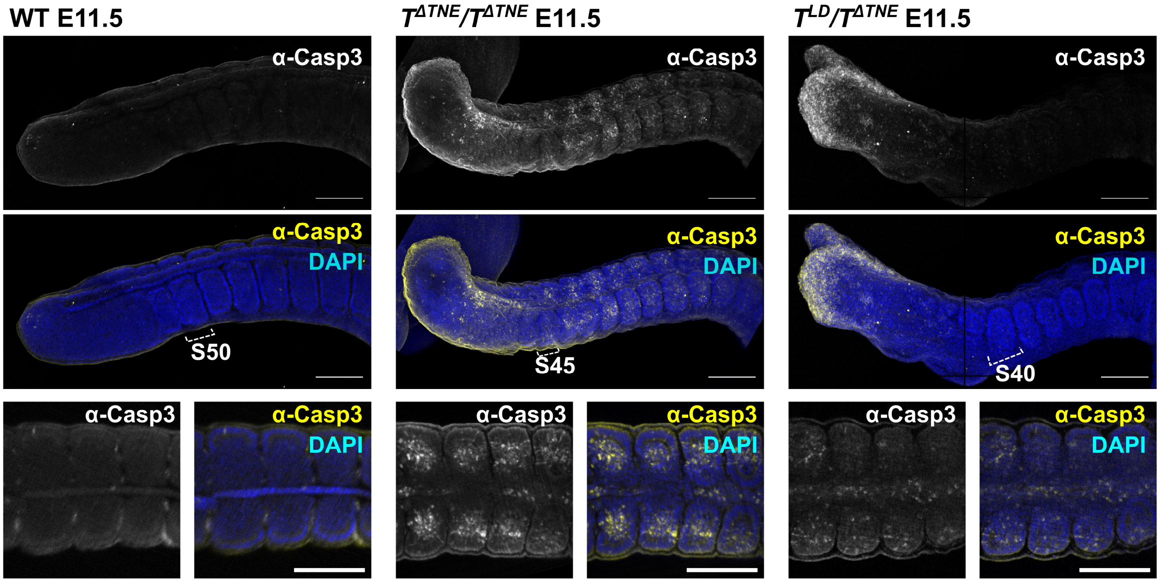

Fig. S7. Mutant tails lacking the notochord undergo apoptosis

Top: Maximum intensity projections of confocal stacks showing wild type (WT) and mutant tails. Extensive Caspase 3 (Casp3) immunofluorescence indicate massive induction of programmed cell death (reviewed in Porter and Jänicke, 1999) in mutant tails compared to wild type. The number of the last formed somite (S) is indicated. Casp3 signal shown as single channel and overlap with DAPI nuclear staining. Bottom: Coronal optical sections acquired by Light sheet microscopy showing strong Casp3 expression in somites and midline cells. Scale bar = 500 µm.

Development • Supplementary information

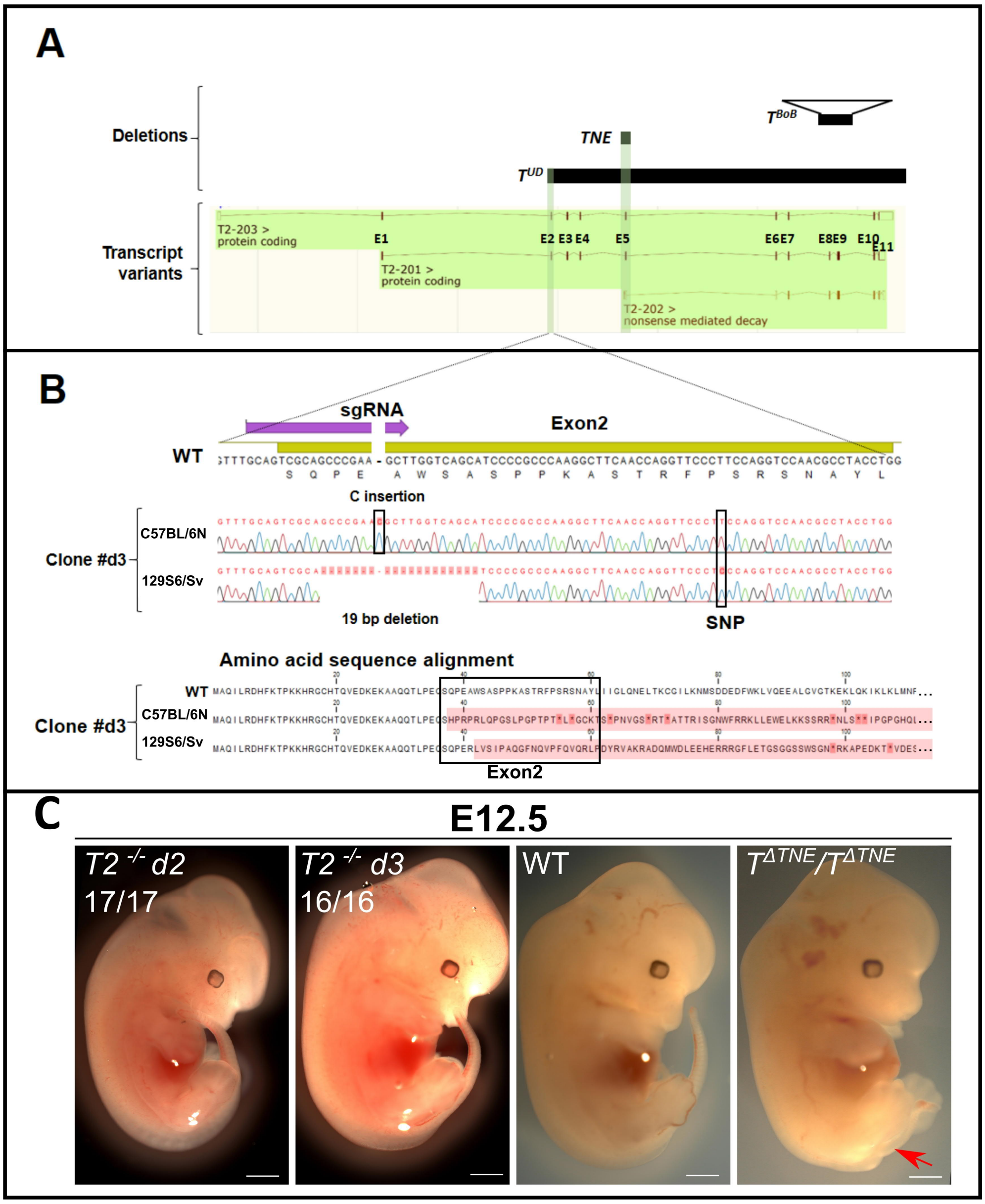

Fig. S8. Disruption of the T2 ORF does not induce a tail phenotype

(A) *T2* transcript variants annotated in the ensemble database. Location: Chromosome 17: 8,355,992- 8,423,513 forward strand (mm10). Top: Mutant genotypes covering exons of the transcript. **(B)** Knock-out strategy using a single CRISPR guide RNA to introduce indels in exon 2. Representative genotyping of one clone. Top: Sanger sequencing of subcloned PCR fragments. Because a hybrid cell line is used, the C57BL/6N and 129S6/Sv alleles were discriminated via a short nucleotide polymorphism. Bottom: Amino acid sequence alignment. Frameshift sequence highlighted in red, stop codons indicated by red asterisks. **(C)** E12.5 Embryos generated via tetraploid aggregation. *T2* -/- does not recapitulate the axial truncation phenotype of $T^{\Delta TNE}/T^{\Delta TNE}$ pointed out by the red arrow. Number of analyzed embryos with the same morphology indicated for *T2* $^{-/-}$ two independent clones (d2 and d3). Scale bar = 1 mm.

Development • Supplementary information

Table S1. List of oligonucleotides used for cloning and PCR

Name	5'-3' Sequence	Purpose
T-dup-u_F1	CCTTTCCCCCTTGATCAC	T^{CD} CRISPR genotyping PCR
Noto_rec_H2B-mCherry_fw	CTCCCATTGAGCTCCTTGCACAGCCTGGGAGGTCCCCTCAGGGTCGCGCAATGCCAGAGCCAGCGAAGTC	BAC reporter recombineering PCR
Noto_rec_H2B-mCherry_rv	GGGCGCAGGCTCCCGGGCTGGACCTGAGTGCCTGAGGGAGCAGGGCTGGATTCCCAGTCACGACGTTGTA	BAC reporter recombineering PCR
Noto_PCR_fw	GGCCTCAATCAGCGATGATTAAG	BAC reporter recombineering PCR
Noto_PCR_rv	CTGGACCTGAGTGCCTGAG	BAC reporter recombineering PCR
PciI-Kozak-Venus_F	TTTTTACATGTCCGCCACCATGGTGAGCAAGGGCGAG	Enhancer reporter cloning
PciI-rbpA_R	TTTTTACATGTACGCGTGCAGTCGAGTTCATAAGAGAAGAGGGACAGCTATGACTGGGAGTAGTCAGGAGAGGAGGAAAAATCTGGCTAGTAAAAGATGTAAGGAAAATTTTAGGGATGT	Enhancer reporter cloning
Seq_U6_F	ACTATCATATGCTTACCGTAAC	Sequencing of gRNA vectors
T2_201_E2_g1_top	CACCGTGCAGTCGCAGCCCGAAGCT	T2 Indel CRISPR cloning
T2_201_E2_g1_bot	AAACAGCTTCGGGCTGCGACTGCAC	T2 Indel CRISPR cloning
T-ds_top	CACCGATCAGTATTGGATCCTCGTT	T^{CD} T CRISPR cloning
T-ds_bot	AAACAACGAGGATCCAATACTGATC	T^{CD} CRISPR cloning
T2_us_C1_top	CACCGATGCACGCTCTTAATCTCGG	T^{CD} CRISPR cloning
T2_us_C1_bot	AAACCCGAGATTAAGAGCGTGCATC	T^{CD} T CRISPR cloning
T_ds_R1	ATGAGAGTGCCTGAGGAG	T^{CD} T CRISPR genotyping PCR
T2_us_F	TATTGGGATGCTGTGCTC	T^{CD} T CRISPR genotyping PCR
T-45.5kb_top	CACCGGCTTCCAACTCCAAGGTAA	T^{UD} CRISPR cloning
T-45.5kb_bot	AAACTTACCTTGGAGTTGGAAGCC	T^{UD} CRISPR cloning
T-8kb8000_top	CACCGCAGCGTAGAGATAGCGGCT	T^{UD} CRISPR cloning
T-8kb000_bot	AAACAGCCGCTATCTCTACGCTGC	T^{UD} CRISPR cloning
T-8kb000_R	GTCTGTCCCTGAGATGATG	T^{UD} CRISPR genotyping PCR
T-45.5kb_F	GTGGCCAGCAAAGGGTGT	T^{UD} CRISPR genotyping PCR

Development • Supplementary information

TNE_sg1_top	CACCGGGGGACCTGACACGTCTCC	$T^{\Delta TNE}$ CRISPR cloning
TNE_sg1_bot	AAACGGAGACGTGTCAGGTCCCCC	$T^{\Delta TNE}$ CRISPR cloning
TNE_sg2_top	CACCGCTGGACTCACAGCGAGTTCT	$T^{\Delta TNE}$ CRISPR cloning
TNE_sg2_bot	AAACAGAACTCGCTGTGAGTCCAGC	$T^{\Delta TNE}$ CRISPR cloning
TNE_sg3_top	CACCGACCCGTCACATCGAGCACCA	$T^{\Delta TNE}$ CRISPR cloning
TNE_sg3_bot	AAACTGGTGCTCGATGTGACGGGTC	$T^{\Delta TNE}$ CRISPR cloning
TNE_sg4_top	CACCGTGTGATTTGGAGATTCGGGT	$T^{\Delta TNE}$ CRISPR cloning
TNE_sg4_bot	AAACACCCGAATCTCCAAATCACAC	$T^{\Delta TNE}$ CRISPR cloning

Development • Supplementary information

Table S2. Antibodies used for Immunofluorescence and ChIP

Description	Catalog number	Company	Host Organism	Concentration	Application
α-T	#81694	Cell Signaling Technology	Rabbit	1:250 in PBSTB	IF
α-Sox2	AF2018	R&D	Goat	1:250 in PBSTB	IF
α-Foxa2	sc-6554	Santa Cruz	Goat	1:250 in PBSTB	IF
α-Sox1	AF3369	R&D	Goat	Rabbit	IF
α-Olig2	AF2418	R&D	Goat	Rabbit	IF
α-Nkx2.2	ab191077	Abcam	Rabbit	Rabbit	IF
α-Caspase-3	9662S	Cell signaling Technology	Rabbit	1:250 in PBSTB	IF
Alexa Fluor 488 α-Rabbit	ab150073	Abcam	Donkey	1:250 in PBSTB	IF
Alexa Fluor 647 α-Goat	ab150135	Abcam	Donkey	1:250 in PBSTB	IF
α-T	AF2085	R&D	Goat	2.5µg	ChIP
