# Supplementary material for: A 37 kb region upstream of brachyury comprising a notochord enhancer is essential for notochord and tail development
Source: Development. 2021 Dec 15;148(23):dev200059. doi: 10.1242/dev.200059 (PMC8722351; doi:10.1242/dev.200059)
Supplement: Reviewer comments [file develop-148-200059_review_history.pdf]

A 37 kb region upstream of brachyury comprising a notochord enhancer is essential for notochord and tail development

Dennis Schifferl, Manuela Scholze-Wittler, Lars Wittler, Jesse V. Veenvliet, Frederic Koch and Bernhard G. Herrmann

DOI: 10.1242/dev.200059

Editor: Patrick Tam

Review timeline

Original submission:	28 July 2021
Editorial decision:	18 August 2021
First revision received:	27 October 2021
Accepted:	8 November 2021

Original submission

First decision letter

MS ID#: DEVELOP/2021/200059

MS TITLE: A 37 kb region upstream of Brachyury comprising a notochord enhancer is essential for notochord and tail development

AUTHORS: Dennis Schifferl, Manuela Scholze-Wittler, Lars Wittler, Jesse V. Veenvliet, Frederic Koch, and Bernhard G Herrmann

I have now received all the referees reports on the above manuscript, and have reached a decision. The referees' comments are appended below, or you can access them online: please go to BenchPress and click on the 'Manuscripts with Decisions' queue in the Author Area.

The overall evaluation is positive and we would like to publish a revised manuscript in Development, provided that the referees' comments can be satisfactorily addressed. Please attend to all of the reviewers' comments in your revised manuscript and detail them in your point-by-point response. If you do not agree with any of their criticisms or suggestions explain clearly why this is so.

We are aware that you may currently be unable to access the lab to undertake experimental revisions. If it would be helpful, we encourage you to contact us to discuss your revision in greater detail. Please send us a point-by-point response indicating where you are able to address concerns raised (either experimentally or by changes to the text) and where you will not be able to do so within the normal timeframe of a revision. We will then provide further guidance. Please also note that we are happy to extend revision timeframes as necessary.

Reviewer 1

Advance summary and potential significance to field

This manuscript by Schifferl et al examines a central question in developmental biology, namely how expression of a key developmental regulators is controlled in distinct cell types and spatiotemporal contexts.

© 2021. Published by The Company of Biologists under the terms of the Creative Commons Attribution License (https://creativecommons.org/licenses/by/4.0/).

They focus on Brachyury (T), one of the principal transcription factors that controls embryonic axis elongation in two ways: by 1) influencing axial neuromesodermal progenitor (NMP) maintenance and their differentiation toward presomitic mesoderm and 2) directing notochord specification

Distinct regulatory elements have been shown to mediate T expression in the primitive streak (PS) and the node/notochord respectively and although the ones regulating the former have been well defined, the exact identity of enhancers conferring notochord-specific T expression has been unknown. In this manuscript the authors have tried to address this issue. Using transgenic embryos carrying various T deletion alleles, they identify a 37 kb upstream region that appears to be crucial for T expression/function in the notochord and tailbud. Based on examination of T binding within this region, they further pinpoint a T-bound enhancer element (=TNE) in the adjacent T2 gene, which directs T expression specifically in the node/notochord and late tail bud. Deletion of this region results in considerable notochord defects indicating its important role in T control in notochord progenitors. The authors also provide further evidence based on mutant embryos, suggesting that TNE is not necessary for head process notochord maintenance and pointing to the existence of a second notochord enhancer which is sufficient to compensate for loss of TNE in trunk but not tail notochord cells. Overall, the study provides a significant insight into T function and its regulation. The experiments and data shown are well executed and presented and I think they support the conclusions of the authors.

Comments for the author

I feel that the manuscript would benefit greatly by the inclusion of some additional data that would provide a deeper and more comprehensive understanding of the molecular/signalling/cellular mechanisms underlying the phenotypes observed:
1) Previous studies have indicated that the node/notochord progenitors may serve as a stable niche that is critical for NMP function/maintenance during axis elongation (e.g. Wymeersch et al Development 2019; Edri et al Development 2019). It would therefore be highly informative if the authors included data examining in detail the effect of eliminating/mis-specifying posterior node/notochord progenitors via the TNE-UD deletions on NMP temporal dynamics/numbers/proliferation as well as their cell fate decisions.
2) Linked to the point above: some of the phenotypes observed (e.g. amorphous caudal mass, tail bifurcation) are reminiscent of defects observed in retinoic acid (RA)-treated embryos as well as in T/T embryo chimeras (Padmanabhan, 1998 Reprod Toxicology; Schmidt et al Dev Biol 1997). Moreover, Wnt signalling has been shown to be important in posterior notochord specification, likely at the expense of an endodermal fate (Ukita et al Mech Dev 2009). Do the TNE/UD deletions disrupt the balance of RA/Wnt signalling pathway activities in the affected regions and cell populations?
Examining the expression of components of these pathways would provide a more detailed insight into the cell vs non-cell autonomous effects of the lack of T functionality in the context of notochord development.
3) Final, a more minor point: Zhu et al PNAS 2016 reported that shRNA-mediated T knockdown in notochord cells diverts them toward a neural fate.
Was this also observed in any of the T mutants examined here? It appears that there are ectopic Sox2+ cells present in the location of the notochord in E11.5 tails of TNE-deleted embryos (Fig. 3G)- it would be great if the authors discussed their findings in the light of the Zhu et al findings.

<u>Reviewer 2</u>

Advance summary and potential significance to field

This manuscript addresses the regulation the T(Brachyury) gene in the notochord. Guided by the phenotype of an allele containing an insertion upstream of the T coding region, they identify the 37 kb genomic region upstream of the T transcriptional start site required for expression of this gene in the notochord. Within this region they identify a ~600 bp genomic region highly conserved among amniotes that is bound by the T protein according to ChIP-seq analyses. The authors characterize this fragment by combining reporter assays, with deletion mutants using CRISPR/Cas9. They show that this region, which they call TNE is a bona fide T enhancer responsible for the expression of this gene in the tail notochord. From the analysis of various combinations of mutants, the authors also suggest the existence of an additional notochord enhancer within the 34kb region that partially

© 2021. Published by The Company of Biologists under the terms of the Creative Commons Attribution License (https://creativecommons.org/licenses/by/4.0/).

replaces TNE activity. As TNE corresponds to an exon of a mouse-specific gene known as T2, the authors generated null mutants for this gene to rule out that the phenotypes associated with the deletion of the TNE enhancer from the genome resulted from inactivation of the T2 gene. Regulation of T in the paraxial mesoderm progenitors had been worked out already some years ago but so far we had no information regarding its regulation in the node or the notochord. This work provides some light on this issue. I therefore consider this manuscript important to understand vertebrate development.

Comments for the author

I find this manuscript experimentally very solid and that the conclusions are supported by the data provided in the figures. I only have some minor comments.
1- The authors describe increased apoptosis in the tail region of homozygous mutants for the TNE deletion.
Most of the dying cells are found in the somites, where this enhancer is not expressed. Could the authors explain this effect?
2- Noto-expressing cells, which, as expected, in normal embryos are restricted to the notochord, became dispersed in homozygous mutants for the TNE deletion, invading the area occupied by the paraxial mesoderm. It would be important that the authors offer some explanation for this odd finding. Could this also be connected to the apoptosis observed in the somites of these embryos?
3- From the analysis of the 37 kb upstream region they conclude that ‘it contains control elements essential for tailbud formation, tail outgrowth and proper trunk differentiation’. I do not think that I can agree with the statement about ‘trunk differentiation’ as written in the manuscript, as it would include all trunk structures.
However, from the data presented here, while trunk somites seem to be indeed affected in these mutants according to Fig 1G’ it seems that other components of the trunk, like the lateral mesoderm, are not affected by the mutation. Trunk development in those mutants also seem obvious from the E12.5 embryos shown in Fig.1. I would suggest that they specify that they refer specifically to the trunk axial and paraxial mesoderm (not general trunk differentiation as implied from the statement in the manuscript).
4- In the introduction, they state “Tbx6 and Msgn1 that commit cells to the mesodermal or paraxial mesoderm fate, respectively”. I thought that Tbx6 is involved in paraxial mesoderm and not in the global commitment of cells to mesodermal (eg. lateral plate mesoderm does not seem to be affected in Tbx6 mutants).
5- In my opinion the phenotypes described in E12.5 embryos are not easily observed in the images provided in the figures.
6- There are some “formatting defects”.
6a. In the second paragraph of the results and discussion section they refer to Fig. 1B-C’ and then “ref”. My guess is that they forgot to introduce this reference.
6b. Several of the citations in the reference list are incomplete (eg. lacking page numbers). My guess is that this resulted from incomplete importation of the references into the program they use for adding references. I suggest that they check the accuracy of the citation in the databases associated with those programs.

<u>Reviewer 3</u>

Advance summary and potential significance to field

Schifferl et al dissect the regulatory control of Tbxt, an essential regulator of mesoderm specification and the development of the notochord. They present a series of genomic deletions, engineered with CRISPR/Cas9 that perturb T expression and notochord development to varying degrees in mouse embryos. They identify a novel enhancer located upstream of Tbxt in exon 5 of T2. They reveal that the enhancer is active in notochord cells at caudal levels, and a striking phenotype that results from deletion of the enhancer region alone that appears independent of the role of T2. The authors provide compelling evidence of a novel enhancer regulating notochord development and the transition from trunk to tail.

© 2021. Published by The Company of Biologists under the terms of the Creative Commons Attribution License (https://creativecommons.org/licenses/by/4.0/).

Comments for the author

Overall, I thoroughly enjoyed reading this manuscript. The figures and data presented are of very high quality. The experimental strategy used to identify T-bound sites from notochord progenitors, and efforts taken to confirm enhancer activity, specificity and functional role, are impressive. I have some minor suggestions for an otherwise excellent piece of work.
In figure 4F, the floorplate staining for Foxa2 is not very clear in the UD/UD mutant shown here. It is notable that Chiang et al 1996 report degeneration of the notochord (and in some sections, the notochord is somewhat present despite absence of a floorplate). Comparisons at these axial positions in Figure 4 would benefit from additional markers (such as Nkx2-2, Olig2 and Pax6) to demonstrate whether there is any pronounced effect on DV patterning that may be expected in the UD mutants that fail to generate a proper notochord, relative to TNE double mutants that initially develop a notochord at both cervical and lumbar levels.
The neural tube looks fairly normal in appearance in UD and TNE mutants compared with what appears to be disorganised somites visible in Figure 4C in UD mutants but not TNE.
As the authors point out, the TNE double mutants develop an additional Sox2 positive lumen. Can the authors clarify whether this is a secondary neural tube structure?
As I understand it, the TCd mutants in Figure 3 describe a 63 kb deletion, which is a different deletion to the TCd mutant described in Figure 1 that spans a larger region (stated as from -62kb upstream of T and the inclusion of T as well as 10kb downstream of T). If this is the case, it would be beneficial to use different nomenclature to describe the two different deletions.
The notochord appears to be absent along the length of the axis in the UD/UD mutants in Figure 1F, not just at the trunk of the embryo as stated on page 5.
Please revise the text to reflect this.
On page 6. The TCd/+ embryos (Figure 3D) appear to have abnormal Tbxt and Noto reporter staining already at this stage in the boxed area of the trunk (indicated with 2x) relative to the WT control in B. Please revise the text.
Page 7. The authors state “Only Tcd/TNE embryos also developed severe defects in trunk differentiation presumably due to lack of the notochord (Fig. 3M)”. Could the authors please provide data to support this statement, as it is not clear what they are referring to here.
The compound mutants (Tcd/TNE) display a very striking tail phenotype. This seems to resemble the bifurcated tails previously reported in Gdf11 mutants.
Could the authors comment on this in the discussion.

First revision

<u>Author response to reviewers' comments</u>

Response to reviewer comments:

Reviewer 1 Advance Summary and Potential Significance to Field:

This manuscript by Schifferl et al examines a central question in developmental biology, namely how expression of a key developmental regulators is controlled in distinct cell types and spatiotemporal contexts. They focus on Brachyury (T), one of the principal transcription factors that controls embryonic axis elongation in two ways: by 1) influencing axial neuromesodermal progenitor (NMP) maintenance and their differentiation toward presomitic mesoderm and 2) directing notochord specification

Distinct regulatory elements have been shown to mediate T expression in the primitive streak (PS) and the node/notochord respectively and although the ones regulating the former have been well defined, the exact identity of enhancers conferring notochord-specific T expression has been unknown. In this manuscript the authors have tried to address this issue. Using transgenic embryos carrying various T deletion alleles, they identify a 37 kb upstream region that appears to be crucial for T expression/function in the notochord and tailbud. Based on examination of T binding within this region, they further pinpoint a T-bound enhancer element (=TNE) in the adjacent T2 gene, which directs T expression specifically in the node/notochord and late tail bud. Deletion of this region

© 2021. Published by The Company of Biologists under the terms of the Creative Commons Attribution License (https://creativecommons.org/licenses/by/4.0/).

results in considerable notochord defects indicating its important role in T control in notochord progenitors. The authors also provide further evidence based on mutant embryos, suggesting that TNE is not necessary for head process notochord maintenance and pointing to the existence of a second notochord enhancer which is sufficient to compensate for loss of TNE in trunk but not tail notochord cells. Overall, the study provides a significant insight into T function and its regulation. The experiments and data shown are well executed and presented and I think they support the conclusions of the authors.

RESPONSE: We thank the reviewer for the positive evaluation of our manuscript.

Reviewer 1 Comments for the Author:

I feel that the manuscript would benefit greatly by the inclusion of some additional data that would provide a deeper and more comprehensive understanding of the molecular/signalling/cellular mechanisms underlying the phenotypes observed:

1) Previous studies have indicated that the node/notochord progenitors may serve as a stable niche that is critical for NMP function/maintenance during axis elongation (e.g. Wymeersch et al Development 2019; Edri et al Development 2019). It would therefore be highly informative if the authors included data examining in detail the effect of eliminating/mis-specifying posterior node/notochord progenitors via the TNE-UD deletions on NMP temporal dynamics/numbers/proliferation as well as their cell fate decisions.

RESPONSE:
Our data show that none of the mutants we analyzed caused a total loss of Noto+ notochord progenitors. However, strong reduction of Noto+ cell numbers in the CNH region correlated with a loss of tailbud organization resulting in an arrest of tailbud outgrowth. At this point, though we favor the idea, we cannot be sure that notochord progenitors form a niche for NMPs. In the revised manuscript, at the end of the discussion we have included a sentence discussing the correlation between NPCs and NMP maintenance. We try to collect more evidence in ongoing studies, but hope for your understanding that this question is difficult to address and beyond the scope of this manuscript (a report with strong length limitations).

2) Linked to the point above: some of the phenotypes observed (e.g. amorphous caudal mass, tail bifurcation) are reminiscent of defects observed in retinoic acid (RA)-treated embryos as well as in T/T embryo chimeras (Padmanabhan, 1998 Reprod Toxicology; Schmidt et al Dev Biol 1997). Moreover, Wnt signalling has been shown to be important in posterior notochord specification, likely at the expense of an endodermal fate (Ukita et al Mech Dev 2009). Do the TNE/UD deletions disrupt the balance of RA/Wnt signalling pathway activities in the affected regions and cell populations? Examining the expression of components of these pathways would provide a more detailed insight into the cell vs non-cell autonomous effects of the lack of T functionality in the context of notochord development.

RESPONSE:
We need to distinguish T function in the node/notochord and in NMPs. The amorphous caudal mass obtained in T-UD homozygous mutants apparently consists of proneural cells (see Fig. 1 and S1 of the revised manuscript). Since NMPs lacking T activity differentiate into neural tissue this phenotype can be attributed to the loss of NMPs via NMP differentiation into neural cells followed by axis truncation. Retinoic acid is not required. It is also known that RA antagonizes WNT signaling, which explains the phenotypic similarity between loss of WNT or T in NMPs and active downregulation/suppression of WNT or T in NMPs by RA.

3) Final, a more minor point: Zhu et al PNAS 2016 reported that shRNA-mediated T knockdown in notochord cells diverts them toward a neural fate. Was this also observed in any of the T mutants examined here? It appears that there are ectopic Sox2+ cells present in the location of the notochord in E11.5 tails of TNE-deleted embryos (Fig. 3G)-it would be great if the authors discussed their findings in the light of the Zhu et al findings.

RESPONSE:
In the trunk we found diversion of mutant cells to gut endoderm, in the tail mostly to paraxial

© 2021. Published by The Company of Biologists under the terms of the Creative Commons Attribution License (https://creativecommons.org/licenses/by/4.0/).

mesoderm and gut. We used Sox2, T , Foxa2 as markers to distinguish neural (Sox2), notochordal (T and Foxa2) and gut endoderm cells (Sox2 + Foxa2). However, the ventral neural tube of the trunk also expresses Sox2 and Foxa2. Though the latter does not apply to the neural tube of the tail, we cannot exclude that the ectopic Sox2+ tubular structure resembles the ventral neural tube. Thus, we classified the ectopic Sox2/Foxa2 positive structure in the tail as endodermal or ventral neural tube-like. Space limits preclude discussing this point in the main text, but we refer to Zhu et al. in the legend of Figure S6.

Reviewer 2 Advance Summary and Potential Significance to Field:

This manuscript addresses the regulation the T(Brachyury) gene in the notochord. Guided by the phenotype of an allele containing an insertion upstream of the T coding region, they identify the 37 kb genomic region upstream of the T transcriptional start site required for expression of this gene in the notochord. Within this region they identify a ~600 bp genomic region highly conserved among amniotes that is bound by the T protein according to ChIP-seq analyses. The authors characterize this fragment by combining reporter assays, with deletion mutants using CRISPR/Cas9. They show that this region, which they call TNE is a bona fide T enhancer responsible for the expression of this gene in the tail notochord. From the analysis of various combinations of mutants, the authors also suggest the existence of an additional notochord enhancer within the 34kb region that partially replaces TNE activity. As TNE corresponds to an exon of a mouse-specific gene known as T2, the authors generated null mutants for this gene to rule out that the phenotypes associated with the deletion of the TNE enhancer from the genome resulted from inactivation of the T2 gene. Regulation of T in the paraxial mesoderm progenitors had been worked out already some years ago but so far, we had no information regarding its regulation in the node or the notochord. This work provides some light on this issue. I therefore consider this manuscript important to understand vertebrate development.

RESPONSE:
We would like to thank the reviewer for recognizing the importance of our work.

Reviewer 2 Comments for the Author:

I find this manuscript experimentally very solid and that the conclusions are supported by the data provided in the figures. I only have some minor comments.

1- The authors describe increased apoptosis in the tail region of homozygous mutants for the TNE deletion. Most of the dying cells are found in the somites, where this enhancer is not expressed. Could the authors explain this effect?

RESPONSE:
The apoptosis effect stems from the missing notochord and thus from the lack of the Shh signal. It has been shown previously that Shh mutants undergo apoptosis in tissues depending on the Shh signal, such as the neural tube and the paraxial mesoderm; see for instance Teillet et al. (Development 1998; reference was added).

2- Noto-expressing cells, which, as expected, in normal embryos are restricted to the notochord, became dispersed in homozygous mutants for the TNE deletion, invading the area occupied by the paraxial mesoderm. It would be important that the authors offer some explanation for this odd finding. Could this also be connected to the apoptosis observed in the somites of these embryos?

RESPONSE:
Indeed, notochord progenitor cells in the mutant tail mostly end up in the paraxial mesoderm. Ukita et al. (Mech Dev 2009) have shown by lineage tracing that some Noto+ cells in the wild type tail contribute to paraxial mesoderm. These data suggest that axial and paraxial mesoderm formation in the tail are competitive processes.

3- From the analysis of the 37 kb upstream region they conclude that ‘it contains control elements essential for tailbud formation, tail outgrowth and proper trunk differentiation’. I do not think that I can agree with the statement about ‘trunk differentiation’ as written in the manuscript, as it would include all trunk structures. However, from the data presented here, while trunk somites seem to

© 2021. Published by The Company of Biologists under the terms of the Creative Commons Attribution License (https://creativecommons.org/licenses/by/4.0/).

be indeed affected in these mutants, according to Fig 1G' it seems that other components of the trunk, like the lateral mesoderm, are not affected by the mutation. Trunk development in those mutants also seem obvious from the E12.5 embryos shown in Fig.1. I would suggest that they specify that they refer specifically to the trunk axial and paraxial mesoderm (not general trunk differentiation as implied from the statement in the manuscript).

RESPONSE:
This is a valid point: indeed, not all trunk structures are affected, only those dependent on notochord function, in particular on Shh activity from the notochord and/or floor plate. We have changed the wording accordingly.

4- In the introduction, they state "Tbx6 and Msgn1 that commit cells to the mesodermal or paraxial mesoderm fate, respectively". I thought that Tbx6 is involved in paraxial mesoderm and not in the global commitment of cells to mesodermal (eg. lateral plate mesoderm does not seem to be affected in Tbx6 mutants).

RESPONSE:
We thank the reviewer for spotting this "overstatement". We agree that it is too general and therefore have changed it in the revised manuscript.

5- In my opinion the phenotypes described in E12.5 embryos are not easily observed in the images provided in the figures.

RESPONSE:
We have added sections of E12.5 embryos on Fig. S1 and Fig. 3 to demonstrate the phenotypes more clearly.

6- There are some "formatting defects".

6a. In the second paragraph of the results and discussion section they refer to Fig. 1B-C' and then "ref". My guess is that they forgot to introduce this reference.

RESPONSE:
Yes, there was a mistake. We have corrected it.

6b. Several of the citations in the reference list are incomplete (eg. lacking page numbers). My guess is that this resulted from incomplete importation of the references into the program they use for adding references. I suggest that they check the accuracy of the citation in the databases associated with those programs.

RESPONSE:
Importation of references has been difficult; we corrected several times during submission but in the end some mistakes still remained. We have taken care of this for submission of the revised manuscript.

Reviewer 3 Advance Summary and Potential Significance to Field:

Schifferl et al dissect the regulatory control of Tbxt, an essential regulator of mesoderm specification and the development of the notochord. They present a series of genomic deletions, engineered with CRISPR/Cas9 that perturb T expression and notochord development to varying degrees in mouse embryos. They identify a novel enhancer located upstream of Tbxt in exon 5 of T2. They reveal that the enhancer is active in notochord cells at caudal levels, and a striking phenotype that results from deletion of the enhancer region alone that appears independent of the role of T2. The authors provide compelling evidence of a novel enhancer regulating notochord development and the transition from trunk to tail.

Reviewer 3 Comments for the Author:

Overall, I thoroughly enjoyed reading this manuscript. The figures and data presented are of very

© 2021. Published by The Company of Biologists under the terms of the Creative Commons Attribution License (https://creativecommons.org/licenses/by/4.0/).

high quality. The experimental strategy used to identify T-bound sites from notochord progenitors, and efforts taken to confirm enhancer activity, specificity and functional role, are impressive. I have some minor suggestions for an otherwise excellent piece of work.

RESPONSE:
We would like to thank the reviewer for appreciating our work.

In figure 4F, the floorplate staining for Foxa2 is not very clear in the UD/UD mutant shown here. It is notable that Chiang et al 1996 report degeneration of the notochord (and in some sections, the notochord is somewhat present despite absence of a floorplate). Comparisons at these axial positions in Figure 4 would benefit from additional markers (such as Nkx2-2, Olig2 and Pax6) to demonstrate whether there is any pronounced effect on DV patterning that may be expected in the UD mutants that fail to generate a proper notochord, relative to TNE double mutants that initially develop a notochord at both cervical and lumbar levels. The neural tube looks fairly normal in appearance in UD and TNE mutants, compared with what appears to be disorganised somites visible in Figure 4C in UD mutants but not TNE.

RESPONSE:
We have included Olig2 and Nkx2-2 antibody stainings of wild type and mutant embryos showing a ventral shift of the Olig2 and Nkx2-2 domains in the neck region of T-UD but not T-□TNE homozygous mutant embryos, which is explained by lack of the notochord and an impaired floor plate function or maintenance (Foxa2 is expressed, though lower than in WT) in the former. Stronger neural tube patterning defects were detected in the trunk.

As the authors point out, the TNE double mutants develop an additional Sox2 positive lumen. Can the authors clarify whether this is a secondary neural tube structure?

RESPONSE:
We stained for Sox2, Foxa2 and T protein. The additional Sox2 positive tubular structure in place of the notochord was positive for Sox2, Foxa2, and T protein, but the latter only in the tailbud, not in the more anterior section. Sox2 protein and Foxa2 are co-expressed in the gut (see WT for comparison), but also in the ventral neural tube of the trunk (but not of the tail). Since we cannot distinguish between the two we conclude that the ectopic structure is gut endoderm or ventral neural tube-like.

As I understand it, the TCd mutants in Figure 3 describe a 63 kb deletion, which is a different deletion to the TCd mutant described in Figure 1 that spans a larger region (stated as from -62kb upstream of T and the inclusion of T as well as 10kb downstream of T). If this is the case, it would be beneficial to use different nomenclature to describe the two different deletions.

RESPONSE:
We have changed the designation accordingly.

The notochord appears to be absent along the length of the axis in the UD/UD mutants in Figure 1F, not just at the trunk of the embryo as stated on page 5. Please revise the text to reflect this.

RESPONSE:
Yes, we deleted the word “trunk” in this context.

On page 6. The TCd/+ embryos (Figure 3D) appear to have abnormal Tbxt and Noto reporter staining already at this stage in the boxed area of the trunk (indicated with 2x) relative to the WT control in B. Please revise the text.

RESPONSE:
done

Page 7. The authors state “Only Tcd/TNE embryos also developed severe defects in trunk differentiation presumably due to lack of the notochord (Fig. 3M)”. Could the authors please provide data to support this statement, as it is not clear what they are referring to here.

© 2021. Published by The Company of Biologists under the terms of the Creative Commons Attribution License (https://creativecommons.org/licenses/by/4.0/).

RESPONSE:
We included histological sections in support of the statements.
The compound mutants (Tcd/TNE) display a very striking tail phenotype. This seems to resemble the bifurcated tails previously reported in Gdf11 mutants. Could the authors comment on this in the discussion.

RESPONSE:
We mentioned the Gdf11 KO tail phenotype in the text referring to low expression of T in KO tailbud notochord. However, due to lack of space we could not go further into discussion.

Second decision letter

MS ID#: DEVELOP/2021/200059

MS TITLE: A 37 kb region upstream of Brachyury comprising a notochord enhancer is essential for notochord and tail development

AUTHORS: Dennis Schifferl, Manuela Scholze-Wittler, Lars Wittler, Jesse V. Veenvliet, Frederic Koch, and Bernhard G Herrmann
ARTICLE TYPE: Research Report

I am happy to tell you that your manuscript has been accepted for publication in Development, pending our standard ethics checks. The referee reports on this version and the Editor's note are appended below.

Reviewer 1

Advance summary and potential significance to field

Overall, the study provides a significant insight into Brachyury function and its regulation. The experiments and data shown are well executed and presented and I think they support the conclusions of the authors.

Comments for the author

I think the article has benefited from the inclusion of additional data. I found it very difficult to navigate through the changes as they were not highlighted in the text and my suggestions/comments have been partly addressed so I do not have anything more to add. Nevertheless, I am supportive of the work as it nicely sheds light on Brachyury function and its control during embryonic axis elongation through well-designed and executed experiments.

Reviewer 2

Advance summary and potential significance to field

This manuscript addresses the regulation the T(Brachyury) gene in the notochord. Guided by the phenotype of an allele containing an insertion upstream of the T coding region, they identify the 37 kb genomic region upstream of the T transcriptional start site required for expression of this gene in the notochord. Within this region they identify a ~600 bp genomic region highly conserved among amniotes that is bound by the T protein according to ChIP-seq analyses. The authors characterize this fragment by combining reporter assays, with deletion mutants using CRISPR/Cas9. They show that this region, which they call TNE is a bona fide T enhancer responsible for the expression of this gene in the tail notochord. From the analysis of various combinations of mutants, the authors also suggest the existence of an additional notochord enhancer within the 34kb region that partially replaces TNE activity. As TNE corresponds to an exon of a mouse-specific gene known as T2, the

© 2021. Published by The Company of Biologists under the terms of the Creative Commons Attribution License (https://creativecommons.org/licenses/by/4.0/).

authors generated null mutants for this gene to rule out that the phenotypes associated with the deletion of the TNE enhancer from the genome resulted from inactivation of the T2 gene. Regulation of T in the paraxial mesoderm progenitors had been worked out already some years ago but so far we had no information regarding its regulation in the node or the notochord. This work provides some light on this issue. I therefore consider this manuscript important to understand vertebrate development.

Comments for the author

All my comments to the original version of the manuscript have been properly addressed. I have no additional comments.

<u>Reviewer 3</u>

Advance summary and potential significance to field

Schifferl et al dissect the regulatory control of Tbxt, an essential regulator of mesoderm specification and the development of the notochord. They present a series of genomic deletions, engineered with CRISPR/Cas9 that perturb T expression and notochord development to varying degrees in mouse embryos. They identify a novel enhancer located upstream of Tbxt in exon 5 of T2. They reveal that the enhancer is active in notochord cells at caudal levels, and a striking phenotype that results from deletion of the enhancer region alone that appears independent of the role of T2. The authors provide compelling evidence of a novel enhancer regulating notochord development and the transition from trunk to tail.

Comments for the author

The authors have addressed the points raised.
Please note the enhancer sequence has not been indicated in capital letters in supplementary figure 4 (as suggested by the figure legend).
Page 6 - "The strongest phenotype was observed in TLD/TΔTNE embryos (Fig. 3G)." I believe this is Figure 3H-I

© 2021. Published by The Company of Biologists under the terms of the Creative Commons Attribution License (https://creativecommons.org/licenses/by/4.0/).
